# Understanding the complexities of antibiotic prescribing behaviour in acute hospitals: a systematic review and meta-ethnography

**DOI:** 10.1186/s13690-021-00624-1

**Published:** 2021-07-23

**Authors:** Gosha Wojcik, Nicola Ring, Corrienne McCulloch, Diane S. Willis, Brian Williams, Kalliopi Kydonaki

**Affiliations:** 1 School of Health and Social Care, Edinburgh Napier University, EH11 4BN Edinburgh, UK; 2 Edinburgh Critical Care Research Group, University of Edinburgh, Edinburgh Royal Infirmary, EH16 4SA Edinburgh, UK

**Keywords:** Antimicrobial resistance, Antibiotic decision-making, Prescribing behaviour, Doctors, Acute hospitals, Meta-ethnography, Qualitative synthesis

## Abstract

**Background:**

Antimicrobial resistance poses a serious global public health threat. Hospital misuse of antibiotics has contributed to this problem and evidence-based interventions are urgently needed to change inappropriate prescribing practices. This paper reports the first theoretical stage of a longer-term project to improve antibiotic prescribing in hospitals through design of an effective behaviour-change intervention.

**Methods:**

Qualitative synthesis using meta-ethnography of primary studies reporting doctors’ views and experiences of antibiotic prescribing in hospitals for example, their barriers to appropriate prescribing. Twenty electronic databases were systematically searched over a 10-year period and potential studies screened against eligibility criteria. Included studies were quality-appraised. Original participant quotes and author interpretations were extracted and coded thematically into NVivo. All study processes were conducted by two reviewers working independently with findings discussed with the wider team and key stakeholders. Studies were related by findings into clusters and translated reciprocally and refutationally to develop a new line-of-argument synthesis and conceptual model. Findings are reported using eMERGe guidance.

**Results:**

Fifteen papers (13 studies) conducted between 2007 and 2017 reporting the experiences of 336 doctors of varying seniority working in acute hospitals across seven countries, were synthesised. Study findings related in four ways which collectively represented multiple challenges to appropriate antibiotic medical prescribing in hospitals: loss of ownership of prescribing decisions, tension between individual care and public health concerns, evidence-based practice versus bedside medicine, and diverse priorities between different clinical teams. The resulting new line-of-argument and conceptual model reflected how these challenges operated on both micro- and macro-level, highlighting key areas for improving current prescribing practice, such as creating feedback mechanisms, normalising input from other specialties and reducing variation in responsibility for antibiotic decisions.

**Conclusions:**

This first meta-ethnography of doctors` experiences of antibiotic prescribing in acute hospital settings has enabled development of a novel conceptual model enhancing understanding of appropriate antibiotic prescribing. That is, hospital antibiotic prescribing is a complex, context-dependent and dynamic process, entailing the balancing of many tensions. To change practice, comprehensive efforts are needed to manage failures in communication and information provision, promote distribution of responsibility for antibiotic decisions, and reduce fear of consequences from not prescribing.

**Trial registration:**

PROSPERO registration: CRD42017073740.

**Supplementary Information:**

The online version contains supplementary material available at 10.1186/s13690-021-00624-1.

## Background

The continuing emergence and spread of antimicrobial resistance (AMR) poses a major threat to public health and patient safety due to associated morbidity, mortality and healthcare expenditure [1]. The AMR crisis has been attributed, to a significant extent, to misuse and overuse of antibiotics [2, 3]. A recent study looking at global antibiotic consumption, expressed in defined daily doses (DDD), found that it increased by 65 % (21.1–34.8 billion DDDs) across 76 countries between 2000 and 2015 [4]. Although the increase was largely driven by low- and middle-income countries (LMICs), even within high-income countries, multidrug-resistant organism rates are rising. It is estimated that in the US and Europe alone, infections caused by antibiotic-resistant bacteria lead to at least 50,000 deaths annually, with hundreds of thousands more dying elsewhere across the world [2]. A simultaneous decline in new drug development by the pharmaceutical industry due to reduced financial inducements and challenging government regulatory mechanisms has further compounded the problem [5].

Rising resistance levels and unavailability of newer agents have led to coordinated efforts to implement new national and international initiatives, resume research efforts and minimise the use of currently available antibiotics to preserve their therapeutic effectiveness. These efforts have been underpinned by the concern that by 2050, AMR-related patient deaths will exceed 10 million annually worldwide, with a projected economic cost of 100 trillion US dollars [6]. Despite coordinated efforts, hospitals worldwide currently face significant problems with inappropriate antimicrobial use, as much as 30–50 % of that usage being unnecessary or inappropriate, leading to worse health outcomes [7, 8]. In the UK, despite some progress in primary care, a sustained reduction in total antibiotic prescribing in secondary care has not been observed. Whilst only 20 % of antimicrobial consumption occurs in hospitals, the intensity of use is far higher than in the community. Evidence shows that hospital usage has increased by 6.3 % over the last five years, despite widespread availability of local and national antibiotic prescribing recommendations [9]. This suggests that prescribing guidelines alone are insufficient to change practice and reduce the problem of AMR.

The need for well-designed antimicrobial stewardship (AMS) interventions has never been more critical. The Medical Research Council has long advocated the importance of identifying theory to understand the likely causal processes of change before undertaking the intervention effectiveness stage [10]. The assessment of the likely barriers and facilitators to inform the selection of intervention components is key to that process. However, systematic reviews of strategies employed to reduce inappropriate antibiotic use in hospitals have shown that behavioural and social influences remain underutilised in designing and evaluating AMS interventions [11]. The existing hospital AMS initiatives are not contextually designed or implemented with end-users of different specialties in mind [12]. As the majority of hospital antibiotic prescribing is currently performed by doctors, an in-depth exploration of the determinants that drive their behaviour within that context is crucial to changing that behaviour and enhancing the chances of planned interventions working in a real-world setting [13].

Although a recent Cochrane review provided recommendations on the effectiveness and safety of interventions to improve antibiotic prescribing to hospital in-patients [8], there remains a gap in the evidence-base on what behaviour change strategies work in hospitals, how to implement them and what refinements are needed to tailor the interventions to local contexts [14]. An exploration of the wide-ranging contextual, organisational and interpersonal determinants in antibiotic decision-making and their influence on different groups of prescribers has not received adequate attention.

The Cochrane Qualitative Implementation and Methods Group has increasingly recognised the importance of including qualitative findings within evidence-based healthcare research [15]. Qualitative research is particularly valuable in providing detailed descriptions of human thinking and behaviour in the contexts in which it occurs and capturing the depth and richness of people`s views and experiences of, for example, delivering or receiving health interventions [16]. To date, some related qualitative syntheses have been conducted, such as in prescribing for respiratory infections in general practice [17] and in hospitals globally, including different groups of prescribers [18]. There is a large body of qualitative studies exploring hospital doctors` antibiotic prescribing experiences, but this has not yet been systematically searched for and integrated within a robust qualitative synthesis. Yet, such research would enable knowledge gained from these hospital doctors’ insights to generate new, clinically applicable theory to inform development of a much needed future behaviour change intervention.

## Methods

This study aimed to identify and synthesise qualitative research reporting doctors` views and experiences of the barriers and facilitators to appropriate antibiotic prescribing in acute hospitals and develop a conceptual model identifying how different pressures and dilemmas influence prescribing behaviour. Appropriate antibiotic prescribing was defined as the practice of initiation, monitoring, review and discontinuation of antibiotic therapy concordant with best practice such as guidelines.

### Study design

There are various methods for synthesising qualitative research [19]. Noblit and Hare’s meta-ethnography (ME) is an interpretive method widely used in health research as it systematically analyses multiple primary studies to go `beyond` their participant and author findings to generate new conceptual models or theories through translation of original study findings reciprocally (accounts across studies are comparable) and refutationally (accounts contradict one another) to create a line-of-argument synthesis (where accounts can be drawn together in a new higher-level interpretation) [20, 21].

ME consists of seven overlapping phases: getting started; deciding what studies are relevant; reading studies; determining how studies relate; translating studies into one another; synthesising translations; and expressing the synthesis [21]. Reflecting the need for study transparency, ME methods and findings are reported according to the eMERGe guidance [22] and detailed in Additional file 1.

Phase 1 (getting started): ME was selected as the most suitable approach because of its ability to develop theory and/or conceptual understandings [21]. This approach was in line with this review’s intention to identify components for a future antibiotic prescribing intervention. The study protocol was devised and registered with PROSPERO (CRD42017073740). Ethical approval was not required.

### Search methods and study selection

To help identify relevant studies (Phase 2), we used the SPIDER tool (Table 1) which facilitates searching of qualitative and mixed-method studies [23]. Our detailed search strategy is provided in Additional file 1. We systematically searched 20 electronic databases, including: EMBASE, MEDLINE, PubMed, ScienceDirect, Web of Science and ZETOC from 2007 to 2017. To maximise return, extensive search terminology and relevant synonyms were used, including medical subject headings (MeSH), supplemented by free-text and broad-based terms.

**Table 1:** Search terms identified using the SPIDER tool [23]

**S**ample (hospital clinicians)	Doctor* OR physician* OR clinician* OR medical staff OR health personnel
**P**henomenon of **I**nterest (antibiotic prescribing in acute hospitals)	Antibiotic prescribing OR overprescribing OR misuse OR overuse OR antibiotic stewardship OR resistance OR guideline adherence OR decision-making OR practice behaviour AND hospital* OR acute care OR hospital ward
**D**esign/ **E**valuation/ **R**esearch type (*qualitative)	Qualitative OR focus group* OR interview* OR ethnograph* OR observation*

See Additional file 1 for Hybrid Qualitative Filters

**Fig. 1:**
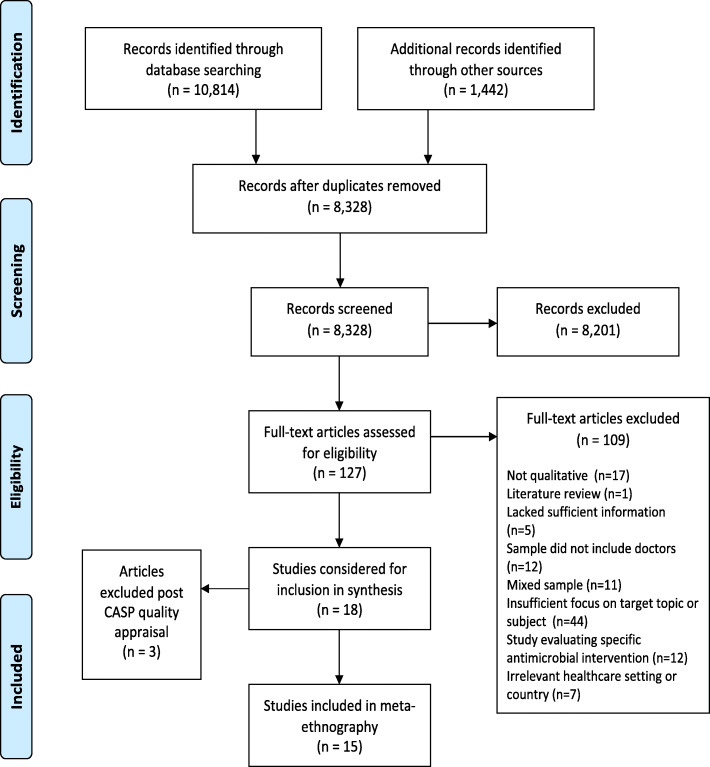
PRISMA diagram [see Additional file 1 for more details]

Identification of qualitative research through electronic databases is challenging [24] so, the online search was supplemented with other methods, including hand-searching of relevant publications, reference screening and citation searching of relevant reviews and included studies. Grey literature sources were searched for, including government reports, audits, conference proceedings and doctoral theses. Potential items for the ME were screened initially by title and abstract and then full text against our inclusion criteria (Table 2) by two reviewers (GW and CM) working independently and then comparing outcomes. Any disagreements were referred to the full team for arbitration. Literature searching outcomes were reported using PRISMA (Fig. 1).

**Table 2:** Study inclusion and exclusion criteria

Inclusion criteria	Exclusion criteria
• Primary research studies reporting doctors` views and experiences of antimicrobial prescribing in acute hospital settings, including adult and paediatrics • Used qualitative methods of data collection (e.g., interviews, focus groups) and inductive analysis (e.g., grounded theory, phenomenological analysis) • Mixed-methods studies only if the qualitative data are discreet and findings reported adequately • Studies carried out in countries considered to have a developed healthcare system according to international classification • Published in English language between 2007 and 2017	• Primary research reporting doctors` views and experiences of prescribing other treatments or other aspects of prescribing e.g., costs, effectiveness. • Research on prescribing antibiotics in other settings e.g., primary care or residential settings • Studies conducted in countries not considered to have a developed health care system^a^ • Sample including prescribers other than acute hospital doctors e.g., general practitioners or nurses • Studies that did not report primary qualitative data collection and analyses e.g., quantitative research, descriptive case studies, commentaries, editorials, reviews. Mixed-methods studies where qualitative data were not reported separately

See Additional file 1for full definitions

### Quality appraisal

Phase 3 (reading included studies): each full-text article was read and re-read. Quality appraisal was conducted by two independent reviewers (GW and CM) based on the Critical Appraisal Skills Programme (CASP) assessment tool, which has been widely used in ME [25, 26]. Quality appraisal is not essential for ME, but it supports close reading of studies and helps assess each study’s contribution to the final synthesis [20].

Data reporting original participant quotes (first-order) and author interpretations of participant data (second-order) were extracted separately into NVivo v11 software. ME requires rich data for synthesis. If extraction revealed a lack of data, for example, few participant quotes, original authors were contacted for further data. If no further information was available, studies meeting our inclusion criteria but lacking data of suitable depth for synthesis were excluded, PRISMA updated (Fig. 1), and such papers were retained for later reflection (Phase 6).

### Analysis

We adopted Toye et al.`s categorisation approach [27], including identifying concepts from qualitative studies, grouping concepts into higher conceptual categories, further re-grouping categories into overarching themes and, developing a line-of-argument that makes sense of the themes. Through constant comparison, studies were first related by findings (Phase 4) to identify ‘concepts’ (key metaphors, phrases and meaningful ideas), the raw data of ME [26], to see how studies compared or not. This was done using the first- and second-order data extracted into NVivo and then organised using Microsoft Excel spreadsheets. As the papers were re-read, additional ideas that arose were noted. Studies were initially grouped by their primary thematic focus into two clusters and then data across the studies were ordered into larger related categories. Continual reference throughout to original studies and conservation of their unique language/terms was critical. The balancing of the developed inter-related larger categories and understanding the way they are influenced by other factors was central to translating the studies into one another, the next stage of ME.

Phase 4 seamlessly led into the analytical process of study translation (Phase 5). Through discussion, similar reported concepts were merged and collapsed into higher conceptual categories pertaining to the same aspects of antibiotic prescribing. First- and second-order constructs (participants` quotes and authors` interpretations) in each study were continuously compared with those in other studies. We used a `hands on` approach, drawing arrows, lines, creating concept maps and matrices. This process was idiomatic and carried out chronologically, starting from the earliest publication. We compared key concepts from paper one with paper two, synthesised them and compared the outcome with paper three, and so on. The interpretations and explanations provided by the original study authors were subsequently compared and translated across papers to achieve a synthesis. Translation of findings was reciprocal where similar concepts (albeit expressed differently) were drawn together and refutational, where contradictory or disconfirming concepts were noted. Where differences were noted, for example, if a study reported different concepts from the others, we returned to full text papers to understand its context, such as whether participants were in a different setting or of different gender. The expanded groupings were then refined and re-arranged for two clusters of studies, first separately and then drawn together until they were considered to explicitly and precisely reflect the synthesised findings. This process enabled us to `go beyond` findings from individual studies, from simple descriptions of the data to developing third-order interpretations [28]. Translation led to the development of overarching themes.

Phase 5 merged into Phase 6 (synthesis of translations), whereby through reflection and discussion we went over and above the developed themes to create a new line-of-argument (LOA), that is our ‘third-order constructs interpretation’, a picture of the findings built on the individual parts of studies [20]. Findings generated during the translation, created spreadsheets, data matrices and our explanations and interpretations provided the foundation for higher analysis. Themes were brought together and matched against original author interpretations and participant quotes from each study to create a new LOA.

Reflection is critical in ME and this was achieved in three ways [26]: team discussions to check accuracy and emerging findings/perspectives; three group consultations across the study with key stakeholders (professionals involved in hospital antimicrobial stewardship and health service users) and, comparing our LOA with findings in studies excluded following quality appraisal to determine whether their inclusion would have altered our final synthesis. Overall, these processes enabled us to reflect on and refine our LOA and propose a new conceptual model of the multi-dimensional nature of medical antibiotic prescribing which we then expressed in our synthesis findings using narrative and visual representation (Phase 7).

## Results

Overall, 12,256 possible references were identified (Fig. 1). Eighteen qualitative papers met our inclusion criteria. Following quality appraisal (Table 3), three papers were excluded due to low quality of their reporting [29–31]. Fifteen papers reporting findings from thirteen studies were finally included in the synthesis [32–46].

**Table 3:** CASP Quality Appraisal

Paper	Decision to retain for Phases 4–6	1. Clear research aims		2. Qualitative methodology appropriate		3. Research design		4. Recruitment strategy		5. Data Collection		6. Reflexivity		7. Ethical Issues		8. Data Analysis		9. Findings		10. Research Value	
		**R1**	**R2**	**R1**	**R2**	**R1**	**R2**	**R1**	**R2**	**R1**	**R1**	**R1**	**R2**	**R1**	**R2**	**R1**	**R2**	**R1**	**R2**	**R1**	**R2**
**Almatar et a.l 2014** [29]	✘	Yes	Yes	Yes	Yes	P	Yes	P	Yes	Yes	Yes	No	No	Yes	Yes	U	U	Yes	Yes	P	Yes
**Almatar 2015** [30]	✘	This is a thesis and is a copy of the above study [29].																			
**Barlow et al. 2008** [31]	✘	P	P	P	Yes	P	P	Yes	Yes	P	Yes	No	No	P	P	No	No	P	Yes	P	P
**Cortoos et al. 2008** [32]	**✓**	Yes	Yes	Yes	Yes	Yes	Yes	Yes	Yes	Yes	Yes	No	P	P	P	Yes	Yes	Yes	Yes	Yes	Yes
**Bjorkman et al. 2010** [33]	**✓**	Yes	Yes	Yes	Yes	Yes	Yes	Yes	Yes	Yes	Yes	No	No	P	P	P	P	P	Yes	P	P
**Broom et al. 2014** [34]	**✓**	P	P	Yes	Yes	Yes	Yes	Yes	Yes	Yes	Yes	P	P	P	P	Yes	Yes	Yes	Yes	Yes	Yes
**Mattick et al. 2014** [35]	**✓**	Yes	Yes	Yes	Yes	Yes	Yes	Yes	Yes	Yes	Yes	P	P	P	Yes	Yes	Yes	Yes	Yes	Yes	Yes
**May et al. 2014** [36]	**✓**	Yes	Yes	Yes	Yes	P	Yes	Yes	Yes	P	P	No	No	P	P	U	U	Yes	Yes	Yes	Yes
**Livorsi et al. 2015** [37]	**✓**	Yes	Yes	Yes	Yes	P	Yes	P	P	Yes	Yes	No	P	P	Yes	Yes	Yes	Yes	Yes	P	Yes
**Livorsi et al. 2016** [38]	**✓**	Yes	Yes	Yes	Yes	Yes	Yes	P	P	P	P	P	P	P	Yes	Yes	Yes	Yes	Yes	Yes	Yes
**Skodvin et al. 2015** [39]	**✓**	Yes	Yes	Yes	Yes	Yes	Yes	Yes	Yes	Yes	Yes	Yes	P	P	Yes	P	Yes	Yes	Yes	P	P
**Broom et al. 2016a** [40]	**✓**	Yes	Yes	Yes	Yes	Yes	Yes	P	P	Yes	Yes	P	P	P	P	P	Yes	Yes	Yes	Yes	Yes
**Broom et al. 2016b** [41]	**✓**	Yes	Yes	Yes	Yes	Yes	Yes	Yes	Yes	P	P	No	P	P	P	Yes	Yes	Yes	Yes	Yes	Yes
**Broom et al. 2016c** [42]	**✓**	Yes	Yes	Yes	Yes	Yes	Yes	Yes	Yes	Yes	Yes	No	No	P	P	Yes	Yes	Yes	Yes	Yes	Yes
**Eyer et al. 2016** [43]	**✓**	Yes	Yes	Yes	Yes	Yes	Yes	Yes	Yes	P	P	No	P	P	P	Yes	Yes	Yes	Yes	Yes	Yes
**Rawson et al. 2016** [44]	**✓**	Yes	Yes	Yes	Yes	P	P	Yes	Yes	P	P	Yes	P	Yes	Yes	Yes	Yes	Yes	Yes	Yes	Yes
**Broom et al. 2017** [45]	**✓**	Yes	Yes	Yes	Yes	Yes	Yes	Yes	Yes	Yes	Yes	No	No	P	P	Yes	Yes	Yes	Yes	Yes	Yes
**Sedrak et al. 2017** [46]	**✓**	Yes	Yes	Yes	Yes	P	Yes	Yes	Yes	P	Yes	Yes	P	P	Yes	P	P	Yes	Yes	Yes	Yes

Key: R1 – reviewer 1; R2 – reviewer 2

Individual decisions: *P:* Partially, *U:* Unable to determine

The included studies were from seven countries across three continents: Australia [34, 42, 45, 46], USA [36–38] and Europe, including the UK [35, 40, 41, 44], Belgium [32], Sweden [33], Switzerland [43] and Norway [39]. Studies were conducted in 43 acute hospitals, including regional, metropolitan, tertiary and secondary care. All included studies involved research carried out in public hospitals and four papers drew the sample from a mix of hospitals (i.e., public, private and federal) [36–39]. Thirteen papers described the hospitals as teaching [32, 35–37, 39–46]. The studies reported the experience of 336 doctors practising across various disciplines from a range of medical and surgical fields. All except three studies [36, 43, 44] provided gender information that included 274 participants, from which 106 (39 %) were women. Not all authors provided details of their study context (i.e., hospital type) and it was not always possible to determine participants` ethnicity, specialty, length of clinical experience, and exact area of medical expertise. Most studies specified participants` level of experience representing a range of seniority (*n* = 14), whilst one study focused specifically on junior (foundation year) doctors [35]. The age of participants ranged between 20 [35] and 70 years [33].

Sample size varied considerably, from 10 [46] to 64 doctors [45]. Data were collected using individual interviews (*n* = 13) [33–35, 37–46], focus groups (*n* = 1) [32] and a mixed-methods approach comprising an online survey and semi-structured interviews followed by an observational study (*n* = 1) [36]. Overall, the studies had an acceptable methodological quality. However, most studies neglected the value of reflexivity (*n* = 12), with only three studies reporting how the authors` social background, location, role, and assumptions may have affected the research process and findings [39, 44, 46].

Characteristics of the 15 papers, including author, year of publication, country/setting, study focus, population, data collection, analytic approach and key findings, are detailed in Table 4.

**Table 4:** Summary of qualitative papers included in the synthesis

Study	Aim(s)	Sample	Data collection & analysis	Key findings
Cortoos et al. 2008 [32]	To determine the opinions and problems concerning the use of a local antibiotic hospital guideline.	1 public tertiary care university teaching hospital	Focus Groups	7 themes reported:
Belgium		22 physicians from internal medicine (7 residents/ 6 staff) and surgery (6 residents/ 3 staff).	Framework Analysis	General attitudes and guideline interpretation; guideline familiarity and awareness; guideline contents and agreement; social influence; multidisciplinary approach, organizational constraints; attitudes about specific interventions.
		Ages: 26-60, 5 females/17 males.		
Bjorkman et al. 2010 [33]	To explore and describe perceptions of antibiotic prescribing among Swedish hospital physicians.	7 acute public hospitals	Semi-structured Interviews	5 main categories of perceptions of hospital antibiotic prescribing and AMR:
Sweden		20 hospital physicians (5 urology physicians, 5 from surgery, 10 from internal medicine).	Phenomenographic Analysis	Prefer “effective” treatment; too uncertain to be restrictive; stuck in the healthcare system; aware and restrictive, but support required; aware, interested and competent.
		Ages: 31-70, 5 females/15 males.		
Broom et al. 2014 [34]	To investigate the experiences of doctors who prescribe antibiotics.	1 acute regional public hospital	Semi-structured Interviews	6 main themes reported:
Australia		30 doctors from: emergency medicine (3), general medicine (4), geriatrics (3), intensive care (2), obstetrics and gynaecology (3), oncology (2), orthopaedics (2), paediatrics (1), renal medicine (2), sexual health (1), surgery (2), urology (1) and infectious diseases (4). House officers (4), registrars (7), advanced trainees (2), consultants/ staff specialists (11), consultants/ senior staff specialists (5).	Thematic Analysis	Everyday sensitivity toward resistance; risk, fear and uncertainty; time, pressure and uncertainty; benevolence and the emotional prerogative; habitus and the internalisation of peer practice norms; hierarchies and the localisation of antibiotic prescribing.
		9 females/21 males.		
Mattick et al. 2014 [35]	To explore the antimicrobial prescribing experiences of foundation year (FY) doctors.	2 public secondary care teaching hospitals	Narrative Interviews	6 overarching themes reported:
UK (England & Scotland)		33 junior doctors (21 FY1 and 12 FY2) working in medical and surgical wards.	Framework Analysis	Personal incident narratives about antimicrobial prescribing; antimicrobial prescribing experiences; systems issues; working relations; educational experiences and needs; process-related data.
		Ages: 20-35, 18 females/15 males		
May et al. 2014 [36]	To explore current practices and decision-making regarding antimicrobial prescribing among Emergency Department (ED) clinical clinicians.	8 acute hospitals, including: 5 private (2 tertiary care and 3 tertiary academic centres), 2 federal and 1 public	Semi-structured Interviews (mixed-methods study)	5 overarching themes reported:
USA		21 clinicians (attending physicians, residents, and mid-level clinicians with at least 2 years of ED experience).	Thematic Analysis	Resource and environmental factors that affect care; access to and quality of care received outside of the ED consult; patient-provider relationship; clinical inertia; local knowledge generation
		No gender documented.		
Livorsi et al. 2015 [37]	To understand the professional and psychological factors that influence physician antibiotic prescribing habits in the inpatient setting.	2 acute teaching hospitals (1 public tertiary care and 1 federal)	Semi-structured Interviews	4 themes reported:
USA		30 inpatient physicians: 10 physicians-in-training (8 internal medicine, 2 internal medicine/paediatrics) & 20 supervisory staff (17 hospital medicine, 3 pulmonary/critical care).	Thematic Analysis	Antibiotic over-use is recognised but generally accepted; the potential adverse effects of antibiotics have a limited influence on physicians' decision-making; physicians-in-training are strongly influenced by the antibiotic prescribing behaviour of their supervisors; reluctance to provide critique, feedback or advice.
		10 female/20 males		
Livorsi et al. 2016 [38]	To assess physician knowledge and acceptance of antibiotic-prescribing guidelines through the use of case vignettes.	2 acute teaching hospitals (1 public tertiary care and 1 federal)	Semi-structured Interviews	3 major themes reported:
USA		30 inpatient physicians: 10 physicians-in-training (8 internal medicine, 2 internal medicine/paediatrics) & 20 supervisory staff (17 hospital medicine, 3 pulmonary/critical care).	Thematic Analysis	Lack of awareness of specific guideline recommendations; tension between adhering to guidelines and the desire to individualise patient care; scepticism of certain guideline recommendations.
		10 female/20 males		
Skodvin et al. 2015 [39]	To investigate factors influencing antimicrobial prescribing practices among hospital doctors.	12 public and 1 private hospitals (3 teaching and 10 non-teaching)	Semi-structured Interviews	6 major themes reported:
Norway		15 doctors from five major medical fields (internal medicine (4), surgery (4), infectious diseases specialists (2), other medical field: oncology, neurology and intensive care), Interns/residents/consultants 2/5/8.	Thematic Analysis	Colleagues; microbiology; national guideline; training; patient assessment; leadership.
		Ages: 25-65, 8 females/7 males.		
Broom et al. 2016a [40]	To identify why inappropriate prescribing trends continue.	1 public teaching hospital	Semi-structured Interviews	3 major themes reported:
UK		20 doctors: 8 consultant, 12 non-consultants from medical (15) and surgical specialty (5).	Framework Analysis	Consumerism and complaints culture; priorities, team dynamics and the medical hierarchy; mythical properties of intravenous antibiotics.
		9 females /11 males.		
Broom et al. 2016b [41]	To explore doctors’ experiences of antibiotic prescribing, and the role of social and institutional factors in influencing the decision-making process.	1 public teaching hospital	Semi-structured Interviews	3 major themes reported:
UK		20 doctors: 8 consultant, 12 non-consultants from medical (15) and surgical specialty (5).	Framework Analysis	Negotiating multiple masters; junior doctors ‘stuck in the middle’ between infectious diseases, clinical microbiology and their supervising team; the dynamics of laboratory vs clinical medicine; the transmission of habit: evidence confronts mentoring, anecdote and experiential learning.
		9 females /11 males.		
Broom et al. 2016c [42]	To explore the potential social dynamics underpinning doctors’ antibiotic use and infection management practices.	1 public regional teaching hospital	Semi-structured Interviews	4 main themes reported:
Australia		30 doctors from emergency medicine (3), general medicine (4), geriatrics (3), intensive care (2), obstetrics and gynaecology (3), oncology (2), orthopaedics (2), paediatrics (1), renal medicine (2), sexual health (1), surgery (2), urology (1) and infectious diseases (4). Sample included house officers, registrars, advanced trainees, consultants/staff specialists and consultants/senior staff specialists.	Thematic Analysis	Contesting ‘best’ practice: risk and ambivalence; ‘fear of losing them’ and the role of patient vulnerability; intra-professional and workplace context; ‘craft groups’ and the perpetuation of localised norms.
		9 females /21 males.		
Eyer et al. 2016 [43]	To determine reasons for using antibiotics to treat asymptomatic bacteruria in the absence of a treatment indication.	1 public tertiary care university teaching hospital	Semi-structured Interviews	5 main themes reported:
Switzerland		21 general medicine physicians: 12 residents/9 senior physicians.	Thematic Analysis	Treatment of laboratory results without considering the clinical picture; physician-centred factors; external factors; lack of attention to detail or analytical thinking, particularly under time constraints; overtreatment due to trivialization of urinary tract infection.
		No gender documented.		
Rawson et al. 2016 [44]	To map out and compare the decision-making processes employed for acute infection management on the hospital wards by non-infection medical specialties and explore any factors that influenced this process.	3 public university teaching hospitals (mix of secondary and tertiary care providers)	Semi-structured Interviews	3 overarching themes reported:
UK		20 physicians (9 consultants, 4 registrars, 2 trainees, 5 junior doctors) from non-infection medical specialties (general internal medicine, such as cardiology, respiratory, and geriatric medicine) and augmented care specialties (haematology and nephrology).	Grounded Theory	Mapping the decision-making process; factors influencing the decision-making process; windows of influence on decision making.
		No gender documented.		
Broom et al. 2017 [45]	To examine how hospital doctors balance competing concerns around antibiotic use and resistance.	2 acute public teaching hospitals (1 regional and 1 metropolitan)	Semi-structured Interviews	2 key themes:
Australia		64 doctors from anaesthetics, emergency, geriatrics, gynaecology, haematology, ICU, infectious diseases, nephrology, oncology, orthopaedics, paediatrics, palliative care, respiratory, sexual health, and surgery.	Framework Analysis	The significance of resistance for the hospital and the role of doctor in perpetuating resistance; overprescribing; easier and without perceived immediate risk.
		27 junior doctors, 37consultants.		
		28 females/36 males.		
Sedrak et al. 2017 [46]	To elucidate potential barriers and enablers to the adherence to antibiotic guidelines by clinicians treating community-acquired pneumonia.	1 public tertiary teaching hospital	Semi-structured Interviews	3 main categories reported:
Australia		10 clinicians from emergency medicine (4), general medicine (4) and infectious disease (2). 5 registrars and 5 consultants.	Thematic Analysis	Knowledge, including familiarity with guidelines; attitudes, including confidence in antibiotic guidelines; behaviour, including documentation and communication, experience and clinical judgement.
		5 females/5 males.		

Studies related (Phase 4) by their focus into two clusters:

- Cluster A – studies that focused on the adherence to antimicrobial guidelines, including the barriers and enablers to uptake and the suboptimal use [32, 38, 46].
- Cluster B - studies describing the experience of antibiotic prescribing with differing levels of emphasis placed on the influences on the prescribers` behaviour, ranging from the drivers of antibiotics prescribing, clinical decision-making to awareness of AMR [33–37, 39–45].

Across clusters A and B, 142 concepts emerged with the resulting 17 higher conceptual categories (HCCs) or `piles` that shared meaning. The reported concepts within each conceptual category are detailed in Additional file 2. From these concepts and HCC, four overarching themes were identified during study translation (Phase 5): (1) Loss of ownership of prescribing decisions, (2) Tension between individual care and broader public health concerns, (3) Evidence-based practice versus bedside medicine, and (4) Diverse priorities between different clinical teams. Themes 1–3 were derived from reciprocal translation (findings were compatible). Theme 4 arose from refutational analysis when it was noted that some translated findings described alternative dissonant perspectives of the same phenomenon. Themes are presented below with narrative exemplars in Additional file 3.

### `Loss of ownership of prescribing decisions`

Many hospital healthcare professionals have a role in antimicrobial stewardship but overall responsibility for antibiotic decisions lies with prescribing clinicians. Many decisions are made by senior clinicians and then enacted by junior doctors. However, during nights and weekends, this arrangement shifts, and junior doctors are often expected to manage complex cases alone and make decisions to prescribe antibiotics on behalf of their senior colleagues, with limited support and feedback available at the time [34–40, 42, 44, 46].

When care delivery happens ‘out-of-hours’, the allocation of prescribing responsibility becomes ambiguous. Although junior doctors are expected to initiate or escalate antibiotics, they are hesitant to question or change decisions of their senior colleagues consequently reporting feelings of disempowerment [35, 41, 42, 44]. De-escalating or stopping treatments is considered a senior medical decision-maker role as this requires professional confidence and experienced clinical judgement. Making an independent clinical judgement is viewed by less experienced doctors as unrealistic, or `*something of a dark art’* [42], highlighting variation in the perceived responsibility for prescribing decisions.

Patients transitioning between hospital wards means that the provision of care takes place in multiple hospital locations and across various professional groups, adding to the complexity. Doctors` rotations, rapid ward rounds, numbers of staff delivering care and patients being cared for `remotely` from their primary medical team compounds the problem, leading to frustration, anxiety and ultimately distancing from engaging with decision-making [33, 35–37, 39, 40, 43, 44]. Lack of awareness of what ultimately happens to the patient and whether the prescribed antibiotic therapy was the correct choice for the patient denies junior doctors the opportunity to learn from occasions when their prescribing decisions had been over-ruled or changed.

There was also a concern that some information handed over to the next shift (or clinical area) is not always acted on and prescribed antibiotics are not reviewed by the subsequent clinical team taking over a patient’s care. Fast-paced clinical environments, error-prone handovers, disjointed information, and cumbersome IT systems present further challenges [32–37, 39, 40, 42–44, 46]. Three studies highlighted that poor documentation of decisions and inconsistencies in monitoring and treatment plans compounded the problem and created a sense of anonymity or `invisibility` of decisions [35, 41, 44]. When reasons for antibiotic prescriptions in clinical patient notes are not documented, clear or easy-to-find, clinicians have to guess whether initial decisions regarding antibiotic choice and rationale was accurate and justified. This incomplete patient information impacts on clinicians` ability to take ownership of antibiotic prescribing decisions.

### `Tension between individual care and broader public health concerns`

In uncertain clinical situations, doctors must make decisions in the presence of multiple and often conflicting objectives. While the ethical principle of a *`good doctor`* is to make decisions based on what is best for the individual patient [34], at the same time, clinicians have a responsibility to consider population-level consequences of overprescribing. On one hand, antibiotic overprescribing is recognised as a serious global concern but, on the other hand, not treating an infection may lead to serious patient complications, even death [33, 34, 37, 40, 42–45], and loss of professional reputation. The abstract reality of future AMR causes internal conflict for the treating clinician facing the concrete reality of the ‘here and now’ - the patient`s clinical status and perhaps pressure from family and patients to ‘do something’. The short-term individual costs (for patients and professionals) have to be constantly weighed up against longer-terms societal gains.

Although clinicians consider AMR and its potentially severe consequences when choosing treatment, the threat of resistance is generally perceived to be a distant or not immediate issue [33, 34, 37, 42, 45]. With the exception of clinicians working within infectious diseases and microbiology departments [33], most participants appeared to downgrade the importance of the problem and its potentially devastating consequences during their prescribing decision-making process. Long-term effects of resistance at the wider community-level are not prioritised, and some degree of overuse of antibiotics to manage immediate patient risks is considered to be allowed and socially acceptable [33, 34, 37, 40, 45].

The risks of over-prescribing to the individual patient tend to be disregarded [45]. Some clinicians consider antibiotics a *`peripheral thing’*, of `*limited concern*` [34] with the threat of AMR as a theoretical problem, which is morally and professionally important but  not necessarily practical [33, 37, 40, 42, 45]. Recognition that individual practice contributes to the emergence of AMR is generally low and some clinicians are *`desensitised`* to the problem [45]. Absence of feedback on juniors` antibiotic prescribing limits the opportunity to identify reasons for the knowledge deficits and improve prescribing practice.

### `Evidence-based practice versus bedside medicine`

Internal reasoning, or the way clinicians make sense of their decisions, plays a significant role in antibiotic prescribing. Prescribing behaviour, which may at first appear as `non-rational` or at odds with the evidence, is in fact a realistic and logical choice at the bedside, where positive patient outcomes, maintaining professional reputation and approval from supervisors take a priority [33–37, 41–45]. The health of individual patients lies at the core of medical professionalism and forms part of their professional identity. Being seen by the patient and/or relatives to be *`doing good`* drives clinicians to prescribe antibiotics for their patient regardless of whether it is evidence-based or not [44]. This internalised logic of over-prescribing is driven by the desire to improve patient condition(s) or at least provide a *`beacon of hope*` [43]. This rationale interplays with the expectations of never missing a diagnosis. Prescribing antibiotic treatment is seen a confirmation that `*at least something has been done*` [38].

In busy hospital environments, professional competence is being constantly evaluated. Decisions about whether to prescribe antibiotics are heavily influenced by fear of consequences for prescribers. Missing a potentially treatable infection could result in serious patient harm. Administering antibiotics or prolonging their use creates a perception of an emotional safety net [33, 34, 36–40, 42–46]. Although experience helps to identify and treat the severely ill patients, *`erring on the side of caution`* and prescribing antibiotics *`just in case`* provides reassurance and is therefore the default option irrespective of grade or experience [37].

Junior doctors report experiences of being criticised and seen by colleagues as incompetent when deciding not to treat [35, 37, 40, 42, 45]; in contrast, conservative antibiotic decision-making is rarely recognised as good practice [43]. Senior doctors’ preferences, expectations and prescribing habits also influence junior doctors’ prescribing decisions. Junior doctors risk facing social disapproval if their decision not to prescribe is at odds with the `social norms` of the hospital [34].

Patient demand, expectations of patients` families and the developing *`consumerism culture*` pose additional pressure [40], resulting in a low threshold for prescribing antibiotics, [33, 34, 36, 37, 39, 41–43, 45, 46]. Fear of patient complaints and of potential lawsuit drives clinicians to adopt defensive medicine approaches and prescribe broad-spectrum antibiotics unnecessarily, irrespective of the healthcare system they work in (public or private). However, external factors, such as patient access to care in the private health system, hinder doctors’ ability to foster AMS. For instance, in the US, the emergency departments disproportionately provide care to low-income and uninsured patients [36]. As a result, doctors must not only account for the clinical scenario but also consider the patient’s ability to obtain follow-up care.

Prescribing according to guidelines offers some reassurance and protection, provided these are evidence-based, up-to-date, easily available, and accessible and that doctors have time to consult them [32–34, 37–39, 41, 43, 44, 46]. Digressing from antibiotic guidelines is rationalised by the potential discrepancies between guidelines and practice. When the individual case of a patient does not `fit` readily into guidelines, clinical judgement must be applied [32, 35, 38, 41, 46].

### `Diverse priorities between different clinical teams`

Multidisciplinary input is essential during hospital in-patient care. However, a multitude of experts are involved in patient care, with different tasks or interventions performed by different professionals, who may have different goals for the patient, which can result in variation of care, including antibiotic use. For instance, diverse priorities are evident in the weighting given to different phases of the antibiotic decision-making process between speciality groups. Despite a common overall approach, emergency department (ED) clinicians and surgical specialities emphasise immediate patient care and infection prevention including initiating antibiotics [32, 36, 40, 45, 46], whilst medical specialities focus on longer-term infection management concerns, including refining/reviewing of initial prescribing decisions and stopping antibiotics [41, 43, 44].

Heightened awareness of sepsis and associated risks and complications culminates in an urgency for surgeons and ED clinicians to commence antibiotics as soon as possible in anyone suspected of having an infection [36, 39]. By contrast, acute care medicine doctors report a common stepwise approach to the decision process surrounding acute infection management, whereby new information is constantly considered in the context of prior knowledge [44] and the use of microbiology test results when selecting antimicrobial therapy is emphasised. Within the same hospital, different clinical teams can have diverging opinions on, and requirements from, guideline content. For example, whilst surgical groups describe a strict interpretation of antibiotic guidelines [32], internal medicine doctors highlight that guidelines are incomplete by promoting a standardised, `one-size fits all*`* approach to antibiotic prescribing [36, 38, 39, 41, 44, 46].

Most clinicians (both genders, across settings and healthcare sectors) recognise the benefits of collaboration, including the availability of a second opinion in the treatment of infections and the support for the improved use of antibiotic prescribing guidelines. However, junior doctors experience difficulties in negotiating prescribing decisions with multiple authoritative figures from across various clinical teams [34, 35, 41, 44]. Effective collaboration and senior support were perceived by junior doctors as key facilitators in remedying deficiencies in practical knowledge of appropriate antibiotic prescribing [33–35, 38–40, 42–44].

Key professional collaborators identified in antibiotic prescribing were microbiology, infectious disease specialists and pharmacy. Infection diseases specialists were recognised as helping hospital doctors in AMR prevention by promoting and encouraging the use of guidelines and appropriate narrow-spectrum antimicrobials during handover meetings and ward rounds [33, 39]. Clinical microbiology colleagues were reported as acting as an important communication channel in infection management [35, 39, 44]. Medical doctors especially described their services and advice as valuable and convenient to access. Although these experts were generally highly approved across medical and surgical fields, the relationship with them varied significantly depending on individual clinicians` interest in infectious diseases [32, 33, 35, 41, 45].

The presence of ward clinical pharmacists generated conflicting opinions. Most clinicians from medical and surgical groups (mostly males representing different levels of seniority) described pharmacists as helpful in discussing and sharing rationales for antibiotic prescriptions and prompting antibiotic review and de-escalation [37, 40, 44]. However, they were perceived by some participants (mostly male physicians from internal medicine) as interference [32].

#### Line-of-argument synthesis*Line-of-argument synthesis*

**Fig. 2:**
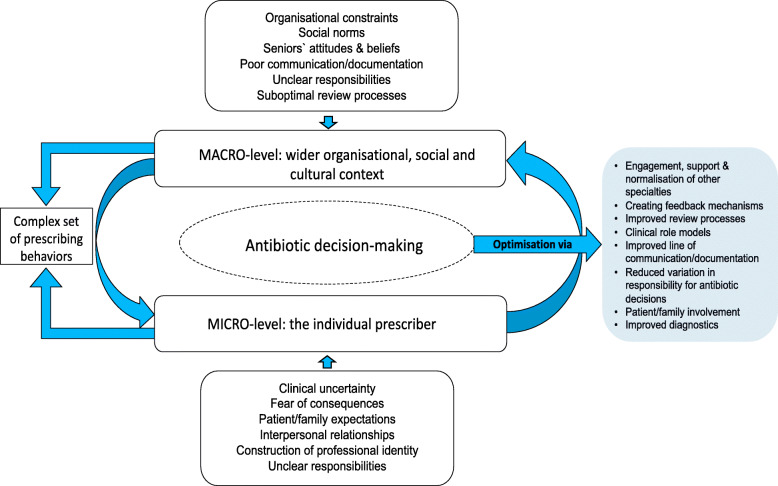
Conceptual model depicting multidimensional nature of antibiotic prescribing in hospital settings

From translation of findings across the 15 studies, a new *line-of-argument* emerged. This final stage in the process of meta-ethnographic analysis (Phase 6) enabled us to develop a higher order interpretation, that is, to generate a conceptual model drawn from, `*but more than the sum of`*, the final themes [21]. Through team reflection and by revisiting the original studies, it gradually became apparent that the four overarching themes overlapped and a more complex nuanced interaction between two micro- and macro-level dimensions of hospital antibiotic prescribing emerged. These two dimensions constantly and simultaneously interacted with each other producing multiple tensions for prescribers and formed the basis for our conceptual model (Fig. 2).

The model illustrates the multidimensional nature of hospital antibiotic decision-making and reflects the array of pressures and dilemmas which need to be balanced by clinicians as they decide their prescribing action(s). This multidimensional nature of antibiotic decision-making describes a complex dynamic and for every clinician, there will be a degree of interdependence between different factors influencing prescribing practice, depending on their level of expertise and ability to tolerate risks for their patient and themselves. The illustrated elements, or factors, will form independent components on one level. However, they are not separate or discreet but constitute an integral part of a whole and will therefore exert a degree of direct or indirect influence on prescribing decisions. These elements coexist, interact, and create a constant dynamic. Both macro (wider social structures, including the norms, standards, social and organisational constraints for human behaviour) and micro (individual behaviours) dimensions feature a complex interplay of influence, authority, and the pursuit of treatment goals. The macro-level structures of hospitals provide the social and cultural setting for healthcare professionals to relate to each other, constantly shaping and influencing micro‐level dimensions that drives individual behaviours and everyday practice.

This unique and evolving dynamic results in the creation of micro-structures of influence, such as internalised logic of prescribing that underpins antibiotic use and drives social interaction with colleagues and patients. An understanding of these contextual drivers of overuse on both macro- and micro-level is fundamental to the development of sustainable interventions to optimise antibiotic use by hospital doctors.

## Discussion

This review is the first to apply an interpretive meta-ethnographic approach and propose a conceptual model to understand the nature of antibiotic prescribing in acute hospitals. The exploration of the challenges to appropriate prescribing in hospitals revealed tensions and uncertainties in antibiotic decision-making by prescribers that occur due to an array of complex organisational and cultural factors. Diversification of priorities between different specialties creates loopholes in the continuity of antibiotic care and treatment. Our review indicates that the transition of patients between wards, busy work environment, high workload, poor documentation and communication and reluctance of junior doctors to question senior colleagues contribute to the partial loss of ownership of antibiotic decisions.

The concept of antibiotic decision ownership does not appear to be highlighted by previous reviews of hospital antibiotic prescribing. It can be argued, however, that when health professionals have a sense of decision ownership, they become personally invested in clinical decisions made for their patients [47]. Although infection management and antibiotic decisions are inherently team-based and interprofessional in nature [48], findings from our ME show that stopping or de-escalating therapy is seen as the responsibility of the consultant or senior specialist. The disparity between expectations of junior clinicians to start but not review and/or stop antibiotics has been previously addressed in a realist review [49], which found that there is a lack of clarity around the specific roles and responsibilities that trainees undertake in relation to antimicrobial prescribing. Communicating an expectation for this group to gain active responsibility for prescribing decisions was suggested as a possible solution to overcome the issue. A recent observational study comparing antibiotic decision-making in acute medical and surgical specialties at a London teaching hospital found that the loss of ownership occurred in the transition of care between the emergency department and inpatient teams specifically [12]. Our ME findings confirm this is the case across different hospital settings and highlights the complexity that arises from each individual`s responsibility for the collective problem of antimicrobial resistance being blurred.

Furthermore, this review has identified inconsistencies in the provision of information between specialties and healthcare professionals. The healthcare system heavily relies on the patient medical records for communication, and safe, and effective care as patients move between wards and their care is handed over between different clinical teams and when staff shifts change. Despite international efforts suggesting that clear documentation of decisions is a key principle in advancing patient safety and improving outcomes [7], the findings show poor documentation of decisions leading to unnecessary continuation of antibiotics as clinicians lack adequate information to make an appropriate decision whether to stop, continue or switch the treatment.

Some studies included in the review appear to indicate that with uncertainty, when an infection is suspected but not proven, the treating clinician will balance immediate clinical risks over long-term population risk. Although commencing antibiotics may be beneficial to the individual, excessive use can increase future AMR and thus be detrimental to the society, a situation known as ‘*the tragedy of the commons*’ [50]. Considering population implications of AMR within bedside antibiotic decisions was viewed by clinicians as difficult. To eliminate concrete clinical concerns, some clinicians will adopt their behaviour accordingly to the culturally accepted norms of the hospital and choose an activity that is perceived as low risk at an individual-level. This fear of consequences heightened by the perception that being conservative in prescribing is not seen as good practice will often lead to prescribing outside of clinical guidelines (either broader spectrum or for longer duration than is clinically indicated), without any clinical benefit to individual patients [18]. Driven by fear of patient deteriorating, an individual`s capacity to adhere to evidence-based practice may be diminished and antibiotic optimisation becomes an absent priority, whilst the risks of over-prescribing to the individual patient tend to be downgraded. This dichotomy between the care recommended in the guidelines and the care provided at the bedside has been reported in earlier works as ‘being on the safe side’ [51].

The ME further highlights the interprofessional nature of antibiotic prescribing and the associated difficulties in negotiating decisions with multiple authoritative figures, including the immediate clinical team and other specialties. Discord in interpersonal relationships was an influencing factor on prescribing decisions, at times leading to poor continuity of care. Inconsistent advice and misunderstanding of roles and responsibilities pertaining to antibiotic decisions were additional barriers to successful collaboration. Challenging decisions of senior colleagues was perceived as unacceptable. The reluctance of junior doctors to question the prescribing decisions can act as an obstacle to gaining a clear understanding of why prescribing choices differ [49]. In such an environment, deferral to the opinion leaders can become the default mode of practice, suppressing valuable input from all members of the team. Yet, qualitative research suggests that doctors tend to feel drawn towards supportive teams and teachers who engage with or inspire them [52]. In environments where senior clinicians are approachable, trust in working relationships increases, allowing junior doctors to raise questions and thus close the communication gap [53, 54]. Examples of good practice included the presence of a clinical pharmacist, infectious disease and microbiology colleagues on the ward prompting the review of antibiotics and acting as effective communication channels.

A collaborative culture fostering a multidisciplinary approach and normalisation of the role of other specialists within the decision-making process are crucial to aid improvements to antimicrobial stewardship [55]. This review demonstrates that the involvement from other specialties in the decision-making depends on the familiarity and acceptance of those colleagues by the senior clinicians. Some junior doctors in these studies described managing interactions with other healthcare professionals as challenging. The `unspoken` yet widely accepted rules on how to manage multidisciplinary dynamics mean that doctors face difficulties steering through the complex system of interrelationships with colleagues that could potentially provide them with assistance. Yet, turning to other specialties for advice can be a source of support outside the scope of medical hierarchy and the immediate clinical team, as junior clinicians experience less fear of appearing ignorant and attracting criticism [56, 57].

Although literature is still lacking with regards to the contexts under which junior doctors feel more able to challenge decisions effectively, quantitative evidence shows that the provision of feedback on the quality of prescribing and direct interaction with prescribers appear to have the most lasting impact on practice [58, 59]. A recent Cochrane review on interventions to improve antimicrobial prescribing practices for hospital inpatients found that interventions that included feedback were more effective than those that did not [8]. Findings of the ME complement the review by showing that creating effective feedback mechanisms and improving communication on prescribing practice has a potential to elicit behavioural change.

In addition, endorsements for the greater integration of other prescribing groups, including pharmacists and nurse prescribers within antibiotic stewardship efforts have already been highlighted by others [60]. For example, lack of partnership with nurses can limit the success of antibiotic stewardship initiatives [61]. Yet, this ME identified an absence of perceived or reported nursing involvement in antibiotic decision-making. This may reflect perceptions about antibiotic prescribing as a process that requires increased knowledge only exclusive to medical professionals with prescribing powers [62], existing and gaps in undergraduate and postgraduate education about antibiotics and AMR [63]. Yet, it remains essential to maximise the contribution of the existing professionals outside infection disease and microbiology towards appropriate use of antimicrobials [64], especially in view of the new Nursing and Midwifery Council guidance highlighting that newly qualified nurses have to be prepared to undertake prescribing training soon after registration [65].

Lastly, antimicrobial prescribing behaviours may vary significantly across different hospital types and be influenced by the types of patients admitted, prescribing patterns and the resources available. For example, a previous study using data gathered from a nationwide survey highlighted major differences in the available resources and implementation of AMS programmes between public and private hospitals in Australia [66]. Moreover, significant differences in antibiotic use remain across different hospital types. In adjusted models, teaching hospitals were associated with lower use of third- and fourth-generation cephalosporins and anti-pseudomonal agents [67]. Although doctors working in private hospitals acknowledged treating more ‘aggressively’ with broader-spectrum antibiotics when patient follow-up was uncertain [36], no major sector-specific drivers of doctors` prescribing behaviour emerged in the synthesis. These findings suggest that antibiotic prescribing across different countries and healthcare systems may be influenced by a similar set of cultural factors [37]. However, given that most studies included in this synthesis were conducted in public teaching hospitals, such as in the UK, the developed model can only be claimed to be representative of that context.

### Strengths and limitations

Locating suitable qualitative studies can be challenging [68] and small-scale qualitative research can be perceived as biased and lacking transferability [69]. However, the number of included studies in the synthesis (*n* = 15) from seven countries and reflecting a breadth of prescribing perspectives was sufficient for conducting ME [20]. The synthesis was carried out in a rigorous way including a large range of databases and grey literature, with continuous input from the experienced research team, undoubtedly reinforcing the credibility of the findings. All stages of the review were checked for accuracy and were grounded in the data by constantly checking the findings against the original studies.

The novelty of this ME is the generation of a higher translation that helps to understand the complexities of hospital antibiotic prescribing decision-making. Although the conceptual model cannot be claimed to be definitive and represent all healthcare practitioners, it offers a unique lens, through which the experiences of doctors can be considered. Meta-ethnography is an interpretative approach and the development of the conceptual model was informed by the review team`s backgrounds and perspectives. The team had considerable expertise in synthesising qualitative research, including experienced health professionals and social scientists with an interest and experience in developing behaviour-change interventions, but none were medical prescribers. GW had been previously involved in projects exploring hospital antibiotic stewardship, NR and BW have extensive experience in conducting ME and NR is a co-author of the eMERGe meta-ethnography reporting guidance. We conducted our research in close affiliation with an NHS hospital trust with advisory input from clinicians during the project. We acknowledge that a different team may have interpreted the included studies differently.

There is currently no gold standard of appraising qualitative studies and including studies with poorly reported methods could produce ME findings lacking credibility [26]. We conducted critical appraisal using the CASP tool, but a different approach of judging the ‘weight of evidence’ of each paper may have been justified. To be included in our synthesis, studies needed to meet a certain degree of methodological transparency. This decision was appropriate as there was a large number of methodologically transparent eligible studies that we could review, and which would enable us to rigorously develop new interpretations and an LOA. After creating our LOA we compared our interpretation and findings against the papers excluded following quality appraisal [29, 31]. This strategy ensured that important insights have not been missed, thus eliminating potential bias. One study raised an issue relating to senior doctors` perception that inappropriate antibiotic prescribing outside guideline recommendations originates with junior doctors [29]. Although this perception did not feature in our analysis, including this paper would not have changed the final synthesis. To enhance the quality of the ME, we updated our database searches in December 2020 and found five studies that met our inclusion criteria [12, 70–73]. However, on critically reading them, we believe that including these studies in our ME would have not refuted our findings but resulted in equivalent meaning.

Not all included studies reported details of participants` characteristics, including gender, ethnicity, level of training, length of experience, and some studies analysed data together for samples drawn from across different clinical settings and healthcare systems. Therefore, it was not always possible to fully identify disconfirming cases between papers or carry out a sub-analysis of different drivers of behaviour based on the sample characteristics and study context. Additionally, five included papers were published by the same researchers research team and although the authors explored prescribing practices in two different countries (Australia and UK), the results may have inadvertently influenced our findings and synthesis [34, 40–42, 45].

The exclusion of studies describing views and experiences of healthcare professionals other than doctors, or where the study population included a mix of healthcare professionals may be contested and a more inclusive approach exploring more diverse perceptions across different clinical groups may have been warranted. However, given that the majority of antibiotics are prescribed by doctors, it was vital to first understand their views and experiences of prescribing practice. The decision was also made to exclude low-income countries to ensure that the theory generated from synthesising primary studies reflects the function of ME and is relevant to the context and setting of the planned antibiotic intervention, that is acute hospitals in well-developed healthcare systems. Including relatively homogenous studies helped strengthen the weight of the conceptual model.

 Finally, to increase the credibility of the review and ensure that the breadth and scope of the data were captured in the synthesis, findings were critically reflected on through regular briefing sessions and workshops with key stakeholders (healthcare professionals involved in hospital antimicrobial stewardship and health service users), providing opportunities to develop and refine ideas and interpretations, and analysed using multiple theoretical perspectives. Although decontextualisation of qualitative findings can be debated [74], the quality and rigour of this review means that it is possible to apply the new conceptual model to a variety of clinical contexts and different groups of healthcare professionals.

### Future practice and research implications

This ME highlights that there is a need to incorporate the influence of the micro- and macro-level elements in the design and delivery of future behavioural-change interventions to optimise antibiotic use in hospital settings. Addressing this complex interaction may be a contributing factor to finding future solutions to the ever-growing problem of AMR and reducing fear of consequences from non-prescribing or stopping antibiotics. Finding new ways of discussing and questioning prescribing decisions between and within clinical teams may be one strategy to mitigate the negative impact of the loss of ownership of decisions and reduce failures in the provision of adequate information. In clinical practice, the influence of senior colleagues could be harnessed by creating role models who act as custodians of professional agendas and create a supportive and open environment that fosters the culture of learning and feedback. The high-level findings presented in this analysis could be further developed for implementation in practice. The insights into ` doctors`conceptualisation of antibiotic use could also have implications for behavioural interventions in other settings, such as primary care or long-term facilities.

The findings in this study concerning the loss of decision ownership may be worth further empirical examination, with a large sample and across a diverse population. It is suggested that future research about promoting effective hospital antimicrobial stewardship focuses on exploring the idea of invisibility of prescribing decisions. Specifically, it would be of value to investigate the diversity of opinions around the roles and responsibilities junior prescribers should undertake in relation to antimicrobial prescribing and how to help overcome uncertainty and fear of consequences. Finding ways to communicate an expectation for this group may foster transfer of active responsibility down the hierarchy ladder. LastlyMoreover, there remains a gap in research concerning the contexts under which junior doctors feel more able to challenge seniors` decisions effectively. Lastly, identifying and comparing inter-hospital factors associated with inappropriate prescribing across different sectors (private vs. public, teaching vs. non-teaching) will help direct future AMS efforts in the specific settings. These areas warrant further investigation.

## Conclusions

This novel ME extends the current evidence-base by providing an understanding of the complexities of hospital antibiotic prescribing. The resulting conceptual framework has the potential to act as the basis for future antibiotic management interventions, exploring clinicians` internal logic of antibiotic prescribing behaviours that goes beyond antimicrobial guidelines and evidence-based practice. Changing ingrained behaviours within a culture or an organisation is undeniably difficult. Yet, improving prescribing practices is essential to minimising the growing public health threat of AMR. It is particularly challenging in acute hospital settings due to the complex relationships between a wide range of stakeholders and multiple teams. Acknowledging this complexity and variability of the hospital contexts and recognising the norms and the ways in which doctors learn to practice will facilitate that change. Healthcare stakeholders can draw on this evidence of *how* and *why* doctors make prescribing decisions to help design and implement more effective antibiotic stewardship interventions in secondary care.

Finally, uncertainty is an unavoidable part of clinical practice and will inevitably persist across all spheres of medicine. Thus, the key dilemma for policymakers and healthcare providers is how to place a higher value on non-prescribing or prescribing narrow-spectrum antibiotics, when available and efficacious, and eliminate a degree of fear while making decisions under uncertain conditions. This ME highlights the need for a more collaborative culture fostering normalisation of the role of other specialists within the decision-making process. The quality of inter-professional relationships between clinicians remains key to achieving this change. Reclaiming the *`*why*`* may act as a positive force to shift the individual risk perceptions and have a positive knock-on effect on changing the culture to open collaboration. This shift will require engagement from senior colleagues, managers and opinion leaders to acknowledge the importance of maximising the explanatory knowledge acquisition.

## Supplementary Information

**Additional file 1.** Details of applied methodology as informed by the eMERGe meta-ethnography reporting guidance. 

**Additional file 2.** Relating studies by reported concepts and developing higher conceptual categories (Phase 4). 

**Additional file 3.** Key emerging themes with exemplar quotes. 

## Data Availability

This systematic review is based on an analysis of a number of published papers which are all referenced within this manuscript. Data supporting our findings is included in the form of the supplementary files listed below.

## Abbreviations

AMR: Antimicrobial resistance
AMS: Antimicrobial stewardship
CASP: Critical Appraisal Skills Programme
HCC: Higher conceptual category
LOA: Line-of-argument
ME: Meta-ethnography
PRISMA: Preferred Reporting Items for Systematic Reviews and Meta-Analyses
SPIDER: Sample, Phenomenon of Interest, Design, Evaluation, Research type
UK: United Kingdom
US: United States
