# Supplementary material for: Understanding the complexities of antibiotic prescribing behaviour in acute hospitals: a systematic review and meta-ethnography
Source: Arch Public Health. 2021 Jul 23;79:134. doi: 10.1186/s13690-021-00624-1 (PMC8299683; doi:10.1186/s13690-021-00624-1)
Supplement: Supplementary file 1 — Additional file 1. Details of applied methodology as informed by the eMERGe meta-ethnography reporting guidance. [file 13690_2021_624_MOESM1_ESM.doc]

**Additional file 1: Details of applied methodology as informed by the eMERGe meta-ethnography reporting guidance**

| **PHASE 1: Getting started** |
| --- |
| There are various methods for synthesising qualitative research, ranging from those aiming to describe or aggregate qualitative findings to those that are more interpretive and generate theory [1]. Meta-ethnography (ME) is a seven phase, theory-generating, interpretive methodology for qualitative evidence synthesis (QES) developed by sociologists [2]. We specifically chose this advanced method as it aims to produce novel interpretations that `go beyond` individual study findings to develop a new conceptual theory or a model whilst preserving the original meanings and contexts of the primary studies [3]. We used ME to generate novel insights and create a conceptual model pertaining to medical antibiotic prescribing behaviours in acute hospitals and thus unveil new and better ways of improving practice.    Our team included experienced health professionals and social scientists (5 white European females and 1 male) with an interest in antibiotic stewardship and expertise in developing behaviour-change interventions. BW and NR had a vast experience in conducting ME and NR was a member of the eMERGe team that developed the ME reporting guidance. As our research was conducted in close affiliation with the NHS hospital trust, we were able to seek advisory input from clinicians during the project.  We also involved patient and public representatives (PPI) in the development of the research protocol for the overall study. The importance of the focus of this meta-ethnography was further endorsed by seeking views of key stakeholders (healthcare professionals involved in hospital antimicrobial stewardship and health service users) who provided insights and challenged the emerging interpretations and thus helped to form the line of argument during three group consultations.  We defined our key terms as follows:  **`Antibiotic`** was defined as any type of a therapeutic agents produced by an organism or made synthetically that selectively destroy or inhibit the growth of micro-organisms [4]. For simplicity, both terms `*antibiotics`* and `*antimicrobials`* were used interchangeably.  **`Antibiotic prescribing`** was defined as the practice of antibiotic use, including initiation, monitoring, review and discontinuation (de-escalation) of antibiotic therapy.  **`Inappropriate *or* suboptimal antibiotic prescribing`** was defined as practice not concordant with local or national guidelines, including: over-prescription (prescribing antibiotics when they are not clinically indicated, e.g. for viral illnesses); omission (when required antibiotics for certain infections are not prescribed); the use of inappropriate dosages (too high or too low); incorrect duration (too short or too long); incorrect selection (mismatch between organisms, for example prescribing a potent broad-spectrum antibiotic when a lower-risk narrow-spectrum agent, which is equally or more effective for treating the same illness/disease, is available); and unnecessary risk (use of intravenous antibiotics when oral forms would be suitable) [5].  **`Acute hospitals`** was defined using the NHS Care Quality Commission definition and referred to hospitals that provide a wide range of specialist care and treatment for patients, including: consultation with specialist clinicians (consultants, nurses, dieticians, physiotherapists and a wide range of other professionals); emergency treatment following accidents; routine, complex and life-saving surgery and specialist diagnostic, therapeutic and palliative procedures [6]. The types of acute hospitals considered was not restricted, and included both children and adult hospitals, and also private, government, university, teaching and tertiary hospitals.  **`Developed healthcare systems`**: as there are currently no standardised quality criteria against which countries healthcare systems performance can be assessed [7], for the purpose of this review, we used an approach employed by Charani et al. [8] and ranked healthcare systems performance using a set of global and most up to date OECD [9] and WHO indicators and adjusted figures for the year 2016 [10]. Based on that, we had 18 countries, including: Australia, Austria, Belgium, Canada, Denmark, Finland, France, Germany, Iceland, Italy, Japan, Netherlands, New Zealand, Norway, Sweden, Switzerland, United Kingdom and United states.  A preliminary search confirmed that there was no QES developed *or* in progress that specifically addressed the topic of interest, and sufficient number of primary studies existed that could be synthesised. This review formed a first stage in developing a theoretical model of a new behaviour-change complex intervention to improve antibiotic use in acute UK hospitals. However, context is crucial not only when designing health interventions but also when evaluating whether that intervention might work in other settings [1,2]. There is an array of disparities between developed and developing countries in terms of healthcare infrastructure, resources, access and provision as well as various social, cultural, political and economic conditions. Therefore, only studies carried out in countries deemed to have a well-developed healthcare system (see definition above) were considered for inclusion. As doctors are responsible for the majority of actual antibiotic prescribing in the UK, it was also essential to first understand this group`s prescribing decisions to change that behaviour. We anticipated that using ME would generate novel insights and aid identification of more effective intervention components.  Finally, we registered our review protocol on PROSPERO systematic review database (CRD42017073740) at <https://www.crd.york.ac.uk/prospero/display_record.php?RecordID=73740>. |
| **PHASE 2: Deciding what is relevant** |
| Details of literature searching, screening and selection process are provided in the methods section of the paper and illustrated in PRISMA diagram (Figure 1).  Our search was informed by SPIDER (Sample, Phenomenon of interest, Design, Evaluation and Research type), designed to facilitate searching of qualitative and mixed-method studies [11]. With an assistance from a University Librarian, we systematically searched 20 electronic databases and grey literature sources, including institutional repositories to search for dissertations and theses, conference proceedings and key organisations to search for reports and audits, such as the Audit Commission, Healthcare Improvement Scotland and The National Institute for Health and Care Excellence (NICE).  The accessed databases included: Academic Search Complete, ASSIA, AMED, BASE, CINAHL, CORE, EMBASE, ERIC, eTHOS, Google Scholar, MedNar, OAIster, OpenGrey, Ovid MEDLINE, ProQuest Dissertations & Theses, PsycINFO, PubMed, ScienceDirect, Web of Science and ZETOC. To maximise return, extensive search terminology and relevant synonyms were used, including medical subject headings (MeSH), supplemented by free-text and broad-based terms. The search strategy consisted of a combination of various search strings, including keywords such as: “antibiotic”, “hospital”, “doctors OR clinicians”, “prescribing”, “choice behaviour”, “decision-making”, “practice patterns” and “guidelines adherence” (see example below for Ovid MEDLINE). The available evidence was then filtered through to identify qualitative studies using hybrid qualitative research filter originally developed by DeJean et al. [12]. To ensure no studies were missed, the search was complemented by searching deep web sources (web pages that are not indexed and cannot be captured by performing standard searches using academic databases). The search was limited to a 10-year period (January 2007 and December 2017) to ensure that views and experiences reflected current policy and practice.  Given the challenges of locating qualitative research, we applied a thorough and transparent methodological search strategy that could be replicated by others using the following:  **1. Electronic search strategy:**  Ovid MEDLINE search strategy including hybrid qualitative filters (1946 - 2017)   1. antibiotics 2. (MM "Anti-Bacterial Agents+/AD/TU") 3. (MM "Bacterial Infections+/DT") 4. TI antibiotic* or antimicrobial* 5. (MM "Drug Resistance, Microbial+") 6. (MM "Medical Staff, Hospital+") 7. (MM "Practice Patterns, Physicians'") 8. (MM "Guideline Adherence") or stewardship 9. (MM "Choice Behavior") 10. (MM "Inappropriate Prescribing") or overprescrib* 11. (MM "Decision Making") OR (MM "Clinical Decision-Making") 12. (MM "Health Knowledge, Attitudes, Practice") 13. (MM "Attitude of Health Personnel") 14. (MM "Physicians+") 15. S2 OR S3 OR S4 OR S5 16. S6 OR S7 OR S8 OR S9 OR S10 OR S11 OR S12 OR S13 OR S14 17. S15 AND S16 18. (MM "Hospitals") or TI hospital* OR AB hospital* 19. S17 AND S18 20. Qualitative Research/ 21. Interview/ 22. Nursing Methodology Research/ 23. (MM "Ethnology") 24. ethnograph$ 25. qualitative 26. ethnonursing 27. phenomenol$ 28. "life stor*" 29. (life stor*).mp. 30. theme* or thematic 31. social construct$ or (postmodern$ or post-struc-tural$) or (post structural$ or poststructural$) or post modern$ or post-modern$ or feminis$ or interpret$).mp. 32. (emic or etic or hermeneutic$ or heuristic$ or semiotic$).af. or (data adj1 saturat$).tw. or participant observ$.tw. 33. "action research" 34. (humanistic or existential or experiential or paradigm$).mp. 35. (field study or studies or research).tw. 36. human science 37. biographical method 38. theoretical sampl$ 39. ((purpos$ adj4 sampl$) or (focus adj group$)).af. 40. ((purpos$ adj4 sampl$) or (focus adj group$)) 41. ((purpos$ sampl$) or (focus group$)) 42. (account or accounts or unstructured or open-ended or open ended or text$ or narrative$).mp. 43. (life world or life-world or conversation analys?s or personal experience$ or theoretical saturation).mp 44. (lived or life adj experience$).mp 45. "cluster sampl*" 46. "observational method$" 47. "content analysis" 48. constant (comparative or comparison) 49. ((discourse$ or discurs$) analys?s) 50. "narrative analys?s" 51. TX ("semi-structured" or semistructured or unstructured or informal or "in-depth" or indepth or "face-to-face" or structured or guide) N3 (interview* or discussion* or questionnaire*) OR TX (focus group* or qualitative or ethnograph* or fieldwork or "field work" or "key informant" or phenomenograph*) 52. (MH "Interviews as Topic") 53. (MH "Focus Groups") 54. (MH "Narration") 55. S20 OR S21 OR S22 OR S23 OR S24 OR S25 OR S26 OR S27 OR S28 OR S29 OR S30 OR S31 OR S32 OR S33 OR S34 OR S35 OR S36 OR S37 OR S38 OR S39 OR S40 OR S41 OR S42 OR S43 OR S44 OR S45 OR S46 OR S47 OR S48 OR S49 OR S50 OR S51 OR S52 OR S53 OR S54 56. S19 AND S55 57. Limiters: Date of Publication: 20070101-20170731; English Language   **2**. **Non-electronic search strategy included:**   - **Reference checking** from key primary studies, studies included in systematic reviews and the studies included in this review. - **Citation pearl searching** of the included studies using the `Cited by` option on Web of Science and Google Scholar, and the `Related articles` option on PubMed and Web of Science. - **Hand searching of key journal**: issues of the American Journal of Infection Control, BMJ Open, The Journal of Antimicrobial Chemotherapy, Clinical Infectious Diseases, Journal of Hospital Infection and Social Science and Medicine to ascertain the completeness of the search strategy. - **Contact with experts:** leading authors in the field were contacted by email for comments and suggestions on key publications, also a list of items that could potentially be included in the review.   We exported the harvested records to Mendeley bibliographic software and screened against eligibility criteria (Table 2) in two stages. After removal of duplicates, all electronic records were initially screened for inclusion by title and abstract by GW. Where title and abstract were equivocal, the full text paper was then read to make a definite decision on the relevance of the study for inclusion in the final synthesis. Full-text evaluation (*n*=127) was conducted first independently, and then collaboratively (GW and CM) to discuss the outcome of the screening process. Where consensus regarding inclusion could not be reached, a third reviewer (NR) within the research team was consulted. When information was unclear or missing from potentially relevant papers, GW emailed the authors and asked for additional information. Out of 17 authors contacted, 14 responded (Bailey, Bjorkman, Broom, Koch, Livorsi, Luetsch, Mattick, May, Pakyz, Pasay, Rawson, Rodriguez, Skodvin and Weeks). Any disagreement regarding eligibility of papers was discussed with the full team. On completion of Phase 2, we identified 18 papers. |
| **PHASE 3: Reading included studies** |
| The 127 papers were then read in full multiple times and quality-appraised using the Critical Appraisal Skills Programme tool [13] by GW and CM. Uncertainties regarding the inclusion were discussed with a third reviewer (NR). Details of quality appraisal are provided in Table 3.  Data extraction was performed verbatim in chronological order. PDF copy of each paper was imported to NVivo V.11 Software and organised separately according to the levels of data (participant quotes (first-order) and original author findings and interpretations of data (second-order). As papers were re-read, direct quotes (first-order constructs) and authors` interpretations (second-order constructs) were coded under separate Nodes. Setting up an additional Node for Original Studies allowed identification of where concepts came from during later phases. We also set up a Node for rejected studies, which we went back to upon completion of the synthesis to check whether important insights have not been missed.  Inadequate, incomplete or ambiguous methodological reporting was a common obstacle to accurately assessing study quality. Following detailed discussion with the research team and reconciliation of the assessment with the third reviewer (NR), consensus was reached to exclude three papers. Two studies were found to lack methodological rigour [14,15], whilst one study was a thesis [16] containing a carbon copy of Almatar et al. [14].  Quality appraisal and data extraction aided becoming familiar with the content of the primary studies and understanding the context and helped us to identify that the quality of studies would have an effect on the contribution of papers to the overall synthesis. For example, paper by Barlow et al. [15] that provided mainly descriptive data offered few insights, while others that included `*thick descriptions*` (conceptually *rich* rather than *descriptive* accounts that included contextual detail) [17] and rigorous analysis, e.g. a paper by Broom et al. contributed more substantively to the analysis [18].  Finally, 15 papers reporting 13 primary studies were considered methodologically sound and were included in Phases 4-7. Four papers were based on two studies. Characteristics of 15 included papers are presented in Table 4. |
| **PHASE 4: Determining how the studies are related** |
| We carried out this phase in several steps. First, we compared the 15 papers by their characteristics, including the author, year of publication, country/setting, study focus, population, data collection and analytic approach (Table 4).  We then related the studies by their findings. GW extracted and coded data line-by-line using NVivo. Codes (metaphors, themes and ideas) were then presented using Microsoft Excel spreadsheets to display recurring concepts (meaningful ideas that developed by comparing particular instances) along with study details [19]. As we progressed, an emphasis was put on refining and merging data into relevant categories. New ideas were allowed to emerge iteratively without *a priori* assumptions. Continual reference to the original studies and conserving their unique language was key in this process. Excel spreadsheets allowed us organisation and comparison of the first-order (participants` quotes) alongside second-order constructs (authors interpretations of those quotes). Extracts were colour-coded to aid identification and differentiation of the studies. As the papers were re-read, a third column was created and additional ideas that arose noted. We then grouped the studies according to thematic focus representing two clusters:   - A=3) papers that focused on the adherence to antimicrobial guidelines, including the barriers and enablers to uptake and the suboptimal use.      - B=12): papers on the experience of antibiotic prescribing with differing levels of emphasis placed on the influences on the prescribers` behaviour from the drivers of antibiotics prescribing, clinical decision-making to awareness of antimicrobial resistance (see findings: Phase 4).   Through constant comparison method, we developed 142 concepts across two clusters of papers. Reflective discussions with the team enabled us to revise, organise and further collapse the 142 concepts into 17 higher conceptual categories (HCCs) that shared meaning. For example, `patient demands` and `perceived patients` preferences` later became a more encompassing HCC of "patient-doctor relationship". This process was time- and labour-intensive but helped to make sense of the data and aid clarity. The HCCs with descriptions are presented in Additional File 2. |
| **PHASE 5: Translating studies into one another** |
| Comparing concepts across 15 papers and regularly discussing the arising ideas seamlessly led into translation of studies into one another. The data contained within each category formed the basis for the analytical process of translation. We arranged all papers chronologically and compared the key concepts from paper one with paper two, synthesised them and compared the outcome with paper three, and so on. The interpretations and explanations provided by the study authors were treated as data, and subsequently compared and translated across the papers to achieve a synthesis. We used a `hands on` approach in this phase, drawing arrows, lines, creating concept maps and matrices. The expanded groupings were then refined and re-arranged for cluster A & B studies, first separately and then drawn together until they were considered to explicitly and precisely reflect the synthesised findings. GW made analytical and reflexive notes during the translations, which were then discussed and challenged by the research group. Thus, our initial broad grouping of ideas was gradually refined by merging and collapsing HCCs into themes. This process enabled us to `go beyond` findings from individual studies, from simple descriptions of the data to developing third-order interpretations [2].  Most studies were similar in focus and allowed reciprocal translation. However, as the studies were compared and translated into one another, some concepts emerged as disparate and they stood in opposition to each other and were thus refutational. This process of translation eventually produced four overarching themes. Whilst themes 1- 3 underwent *reciprocal translation* (findings were compatible), the process of analysis revealed that some of the individually translated findings described alternative or opposing perspectives of the same phenomenon. For example, we observed that there were contradictory concepts related to clinicians` perceptions pertaining to the weighting given to different phases of the antibiotic decision process between speciality groups. This dissonance added a new dimension and a new *refutational* theme 4 was formed (see Findings). |
| **PHASE 6: Synthesising translations** |
| During synthesis of translations, the themes were brought together and matched against authors` interpretations and participants quotes of the respective primary studies. This involved further re-reading of the studies. Third-order analysis was carried out by reflecting on findings from Phase 5 against the spreadsheets and matrices with HCCs, explanations and our interpretations and involved a degree of conceptual innovation. This enabled us to reconceptualise the findings and generate a higher order interpretation of the data. Findings generated during the translation, the created spreadsheets and matrices with the data, and our explanations and interpretations provided the foundation for a higher analysis. Our themes were brought together and matched against original author interpretations and participant quotes of the respective primary studies. As reflection is critical in ME, this was achieved through frequent team discussions [3]. For example, throughout the process, the first author (GW) had her work checked for accuracy (by NR and KK) and emerging findings were presented to the wider team representing different disciplinary perspectives.  On reflection within the team and revisiting the original studies, we observed that the 4 overarching themes overlapped and demonstrated a more complex interaction between the micro- and macro-level dimensions of hospital antibiotic prescribing. For example, we noticed that these two dimensions constantly interact and produce tensions between having to weigh up the imminent risk to the health of an individual patient and the future risk to the health of the society.  Finally, we created a conceptual model or visual representation of the *line-of-argument* (LOA) that was drawn from, `*but more than the sum of`*, the final themes [2] (see Figure 2). Key stakeholders’ involvement: Patient and public representatives were initially involved in the development of the research protocol for the overall study. The importance of the focus of this meta-ethnography was further endorsed by seeking views of key stakeholders (healthcare professionals involved in hospital antimicrobial stewardship and health service users) who provided insights and challenged the emerging interpretations and thus helped to form the line of argument during three group consultations. |
| **PHASE 7: Expressing the synthesis** |
| Findings of this review are presented as narrative, a new conceptual model, supporting tables and supplementary material.  The anticipated audience for this synthesis are: healthcare professionals and managers, professional bodies, policy-makers and those responsible for designing antimicrobial stewardship interventions who may value the practical implications of the findings and also researchers who may be interested in the methodology. This worked example also provides a hypothesis, which other researchers may wish to test.  **Limitations:** see Discussion section of the paper. This review was led by a novice researcher aiming to identify theoretical elements of a new behaviour-change complex healthcare intervention to improve antibiotic use within acute UK hospitals as a first stage of her PhD. However, we resolved this issue through regular meetings and the experienced research team challenged the researcher`s interpretations by expressing alternative views, which ultimately added rigour to the research findings. Although the expertise in synthesising qualitative research was vast among the team members, ME is an interpretative approach and the development of the conceptual model was inevitably driven by the research team`s backgrounds and based on their subjective interpretations. We acknowledge that a different team may have drawn different conclusions.  Due to practical issues (i.e. a large number of eligible studies to work through, also the time and resource-based constraints of a PhD project), the emphasis was placed on the development of new interpretations and a LOA in a rigorous manner rather than producing an exhaustive summary of all studies. Therefore, a threshold to the studies methodological standards was applied. A different approach of judging the ‘weight of evidence’ of each paper ensuring that only studies that provided the conceptual richness and `thick accounts` of doctors` lived experiences were included in the final synthesis may have been justified. However, considering that there is currently no gold standard on appraising qualitative studies, the concern was that including studies with poorly reported methods may produce findings lacking credibility [17]. We therefore decided that the reported methods had to meet a certain degree of methodological `soundness` before inclusion in the synthesis. The critical appraisal using CASP tool was judged appropriate for that purpose [19,20].  Although qualitative themes were a preferred method of displaying findings in the majority of the studies, the degree of clarity differed considerably, from simple classification of findings to meaningfully described theme structures. The blurriness and ambiguity in labelling of some of the themes required a degree of `deciphering` to enable coherent analysis. However, we ensured throughout the process that we preserved the studies unique and original language.  The exclusion of studies describing views and experiences of healthcare professionals other than doctors, or where the study population included a mix of healthcare professionals may be contested and a more inclusive approach exploring more diverse perceptions across different clinical groups may have been warranted. However, given the majority of UK hospital antibiotic prescribing is currently performed by doctors, it was important to first understand this group`s views and experiences of prescribing practice. We also made the decision to exclude low-income countries to ensure that the conceptual theory generated from synthesising primary studies reflects the function of ME and is relevant to the context and setting of the planned antibiotic intervention in the UK. However, the number of included studies in the synthesis (n=15) encompassed the desired criteria, and although not large, it enabled a thorough examination of the phenomenon of interest [17].  Finally, five papers included in the synthesis were published by the same research group and although the authors explored prescribing practices in two different countries (Australia and UK), the results may have inadvertently impacted on the findings of the synthesis.  **Strengths:** The novelty of this meta-ethnography is the generation of a higher translation that helps to understand doctors` experiences of antibiotic prescribing in acute hospital settings.  Although the conceptual model cannot be claimed to be definitive and represent all healthcare practitioners, it offers a unique lens, through which the experiences of doctors` antibiotic prescribing can be considered. The synthesis was carried out in a rigorous and systematic way including a large range of databases and grey literature with a continuous input from the experienced research team, undoubtedly reinforcing the credibility of the findings. All authors had a vast experience in conducting and synthesising qualitative research, whist NR and BW had a special expertise in using ME. NR was involved in developing the ME reporting guidance as part of the eMERGe project to increase the transparency and completeness of the reports [3].  There is little published guidance on updating a meta-ethnography and there is no set time interval after which a meta-ethnography becomes out-of-date [21]. Redoing a new overarching ME or `knocking down and rebuilding the house` could potentially change the findings of the original meta-ethnography [22]. To enhance the quality of the ME, we repeated database searches in December 2020 and found five studies that met our inclusion criteria. However, we believe that including the studies in final analysis would have not refuted our findings but resulted in equivalent meaning.    To increase credibility of the review and ensure that the breadth and scope of the data are captured in the synthesis, findings were reviewed and discussed within the research team through regular briefing sessions providing opportunities to reflect on developed ideas and then refine and analyse interpretations using multiple theoretical perspectives. Although decontextualization of qualitative findings can be debated among methodologists, the quality of this review and rigour applied through all the stages means that it is possible to transfer this *`collective consciousness`* of antibiotic prescribing practice beyond the contextual boundaries and apply the new conceptual model within the broader context of healthcare research that requires identification of both social and clinical dimension[23]. The importance of the focus of this meta-ethnography was further endorsed by seeking views of key stakeholders (healthcare professionals involved in hospital antimicrobial stewardship and health service users) who provided insights and challenged the emerging interpretations and thus helped to form the line of argument during three group consultations.  The key methodological strength of the synthesis is that after creating the LOA, we attempted to interpret the findings against the papers rejected during quality appraisal. This strategy ensured that important insights have not been missed, thus eliminating potential bias and adding to the credibility of the findings. The Almatar et al.`s study [14], which included interviews conducted with eight ED doctors in an Australian hospital (no gender information provided), raised an issue that was not captured in our review relating to senior doctors` perception that inappropriate antibiotic prescribing outside guideline recommendations originates with junior doctors, as opposed to the views expressed by juniors who believed that they always seek advice. A dichotomy between clinicians` views and beliefs and the actual practice is a phenomenon often reported in the literature. Although this perception did not feature in our analysis, including this paper would not have changed the outcome of the synthesis process.  Lastly, the uniqueness of this work lies in the translation process that employed a combination of reciprocal and refutation analysis, which facilitated conceptual innovation that went above and beyond those found in individual studies. The commitment to include refutational data in the synthesis - cases that are exceptions or outliers – helped to enhance the understanding through the development of a LOA. The refutational translation acted as a reminder not to seek similarity alone and to question why some concepts `fit` better than others [24]. |

**References:**

[1] France EF, Ring N, Thomas R, Noyes J, Maxwell M, Jepson R. A methodological systematic review of what’s wrong with meta-ethnography reporting. BMC Med Res Methodol. 2014;14:119. https://doi.org/10.1186/1471-2288-14-119.

[2] Noblit GW, Hare RD. Meta-ethnography: Synthesizing qualitative studies. California: Sage Publications Ltd; 1988. https://doi.org/10.1097/00005053-199007000-00016.

[3] France EF, Cunningham M, Ring N, Uny I, Duncan EAS, Jepson RG, et al. Improving reporting of meta-ethnography: The eMERGe reporting guidance. BMC Med Res Methodol: 2019;19:25. https://doi.org/10.1186/s12874-018-0600-0.

[4] Brunton LL, Chabner BA, Knollmann BC. The Pharmacological Basic Of Therapeutics. The McGraw-Hill Companies Inc; 2011.

[5] Monnier AA, Eisenstein BI, Hulscher ME, Gyssens IC. Towards a global definition of responsible antibiotic use: Results of an international multidisciplinary consensus procedure. J Antimicrob Chemother. 2018;73(6):vi3-vi16. https://doi.org/10.1093/jac/dky114.

[6] Care Quality Commission. How CQC regulates: NHS and independent acute hospitals. Provider handbook. 2015.

[7] Papanicolas I, Kringos D, Klazinga NS, Smith PC. Health system performance comparison: New directions in research and policy. Health Policy. 2013;112:1–3. https://doi.org/10.1016/j.healthpol.2013.07.018.

[8] Charani E, Edwards R, Sevdalis N, Alexandrou B, Sibley E, Mullett D, et al. Behavior change strategies to influence antimicrobial prescribing in acute care: A systematic review. Clin Infect Dis. 2011;53:651–62. https://doi.org/10.1093/cid/cir445.

[9] Organisation for Economic Co-operation and Development. OECD Health Statistics - OECD iLibrary. OECD Heal Stat. 2018. https://doi.org/10.1787/health-data-en.

[10] World Health Organization. Global Health Expenditure Database. 2018. https://apps.who.int/nha/database.

[11] Cooke A, Smith D, Booth A. Beyond PICO: The SPIDER Tool for Qualitative Evidence Synthesis. Qual Health Res. 2012;22:1435–43. https://doi.org/10.1177/1049732312452938.

[12] DeJean D, Giacomini M, Simeonov D, Smith A. Finding Qualitative Research Evidence for Health Technology Assessment. Qual Health Res. 2016;26:1307–17. https://doi.org/10.1177/1049732316644429.

[13] Critical Appraisal Skills Programme. CASP Qualitative Checklist. Casp 2018. ttps://casp-uk.net/wp-content/uploads/2018/01/CASP-Qualitative-Checklist-2018.pdf. Accessed 21 Feb 2018.

[14] Almatar MA, Peterson GM, Thompson A, Zaidi STR. Factors influencing ceftriaxone use in community-acquired pneumonia: Emergency doctors’ perspectives. Emerg Med Australas. 2014;26:591–5.

[15] Barlow G, Nathwani D, Myers E, Sullivan F, Stevens N, Duffy R, et al. Identifying barriers to the rapid administration of appropriate antibiotics in community-acquired pneumonia. J Antimicrob Chemother. 2008;61:442–51.

[16] Almatar MA. Implementation and evaluation of tailored intervention strategies to influence antibiotic prescribing for community-acquired pneumonia. PHD Thesis; University of Tasmania. 2015. https://eprints.utas.edu.au/22746/ Accessed 11 Jan 2018

[17] Campbell R, Pound P, Morgan M, Daker-White G, Britten N, Pill R, et al. Evaluating meta-ethnography: systematic analysis and synthesis of qualitative research. Health Technol Assess. 2011;15:1–164. https://doi.org/10.3310/hta15430.

[18] Broom A, Broom J, Kirby E. Cultures of resistance? A Bourdieusian analysis of doctors’ antibiotic prescribing. Soc Sci Med. 2014;110:81–8. https://doi.org/10.1016/j.socscimed.2014.03.030.

[19] Toye F, Seers K, Allcock N, Briggs M, Carr E, Barker K. Meta-ethnography 25 years on: challenges and insights for synthesising a large number of qualitative studies. BMC Med Res Methodol. 2014;14:80. https://doi.org/10.1186/1471-2288-14-80.

[20] Atkins S, Lewin S, Smith H, Engel M, Fretheim A, Volmink J. Conducting a meta-ethnography of qualitative literature: lessons learnt. BMC Med Res Methodol. 2008;8:21. https://doi.org/10.1186/1471-2288-8-21.

[21] France EF, Wells M, Lang H, Williams B. Why, when and how to update a meta-ethnography qualitative synthesis. Syst Rev 2016;5(44):1-12. https://doi.org/10.1186/s13643-016-0218-4.

[22] McCann S, Campbell M, Entwistle V. Recruitment to clinical trials: A meta-ethnographic synthesis of studies of reasons for participation. J Heal Serv Res Policy. 2013;18(4):233-241. https://doi.org/10.1177/1355819613483126.

[23] Friberg F, Dahlberg K, Petersson MN, Öhlén J. Context and methodological decontextualization in nursing research with examples from phenomenography. Scand J Caring Sci. 2000;14:37–43. https://doi.org/10.1111/j.1471-6712.2000.tb00559.x.

[24] Pilkington H. Employing meta-ethnography in the analysis of qualitative data sets on youth activism: a new tool for transnational research projects? Qual Res. 2018;18:108–30. https://doi.org/10.1177/1468794117707805.
