# Supplementary material for: Understanding the complexities of antibiotic prescribing behaviour in acute hospitals: a systematic review and meta-ethnography
Source: Arch Public Health. 2021 Jul 23;79:134. doi: 10.1186/s13690-021-00624-1 (PMC8299683; doi:10.1186/s13690-021-00624-1)
Supplement: Supplementary file 2 — Additional file 2. Relating studies by reported concepts and developing higher conceptual categories (Phase 4). [file 13690_2021_624_MOESM2_ESM.docx]

**Additional File 2: Relating studies by reported concepts and developing higher conceptual categories (Phase 4)**

| **Higher conceptual category for Cluster A studies with description** | **Concepts included in this category** | **Papers that include 1^st^ order constructs** | **Papers that include 2^nd^ order constructs** |
| --- | --- | --- | --- |
| 1. **Factors that enable and hinder adherence to antimicrobial guidelines**   Barriers to guideline-concordant practice include familiarity and complacency in accessing guidelines, poor or passive guidelines distribution, content not tailored to different specialties and areas, and different guideline sources. Only up-to-date guidelines are viewed as trustworthy. Facilitators to following guidelines include high awareness and acceptance of guidelines, active distribution, accessibility and user-friendly format. | - Distribution & accessibility | 1, 14 | 1, 7, 14 |
|  | - Clarity & interpretation | 1 | 1 |
|  | - Content & agreement | 1, 7, 14 | 1, 7, 14 |
|  | - One size doesn`t fit all | 1, 7, 14 | 1, 7, 14 |
|  | - Need for a cookbook approach | 1 | 1 |
|  | - Familiarity, awareness & acceptability | 1, 7, 14 | 1, 14 |
|  | - Need for evidence-based guidelines | 1, 7 | 1, 7, 14 |
|  | - Guidelines are no dogma but an instrument | 1, 7, 14 | 7 |
|  | - Scepticism around guidelines | 7, 14 | 7, 14 |
|  | - Stringency of applying guidelines | 1, 7 | 14 |
|  | - Different requirements between clinician groups for guideline contents | 1 | 1, 7, 14 |
|  | - Perceived effective strategies to improve guidelines concordance | 1, 14 | 1, 7, 14 |
| 1. **Tension between individualising patient care and adhering to standardised recommendations**   Tension exists between complying with guidelines that only work for standardised patients and the desire to individualised patient care. Clinicians have to rely on their knowledge and experience and tailor care to the specific conditions of the patient. Guidelines are perceived to be in conflict or at odds with the `objective evidence` clinicians collect at the bedside. | - Need to `do something` | 7 | 7 |
|  | - Not to treat is `tough to swallow` | 7 | 7 |
|  | - Stopping antibiotics is nerve wracking | - | 7 |
|  | - Guidelines in conflict with the `objective evidence` | 7, 14 | 7 |
|  | - Fear of missing something | 7 | - |
|  | - Offering antibiotic is easier than withholding treatment | 7 | 7 |
|  | - Modifying guidelines in favour of clinical judgement | 7, 14 | 7, 14 |
|  | - Experience gets in the way of following guidelines | 7 | 7, 14 |
| 1. **Navigating a patchwork system of insufficient healthcare resources**   Busy working environments, disparate prescribing practices between hospitals and wards, poor communication, deficient information transfer, suboptimal follow-up of cultures and treatments are a source of confusion for the less experienced doctors. | - Differences in practice cause confusion | 1 | 1 |
|  | - Documentation, information transfer and patient follow up | 1, 14 | 1, 14 |
|  | - Working pressures & time constraints | 1, 14 | 1, 14 |
|  | - Communication | 1, 14 | 14 |
| 1. **Multidisciplinary collaboration**   Clinicians value multidisciplinary collaboration, including contact and advice from AMS services. Opposing views between clinical subgroups exists towards the presence of clinical pharmacists on the ward. Microbiology specialists are viewed to have lower acceptance than infectious disease. | - Interpersonal relationships | 1, 14 | 1, 14 |
|  | - Role of other specialties (pharmacists, microbiologists and ID colleagues) | 1, 14 | 1, 14 |
|  | - Nurses` influence | - | 1 |
|  | - Involvement of AMS | 14 | 14 |
| 1. **Balancing hierarchy and autonomy**   Senior clinicians are considered as opinion leaders and role models as their practice strongly determines the subsequent prescribing behaviour of junior doctors. This hierarchical and apprentice-based model of medical prescribing is a prominent influence in a hospital. | - Role models | 1, 14 | 1, 14 |
|  | - Apprentice-based model of medical prescribing | 14 | 14 |
| **Higher conceptual category for Cluster B with description** | **Concepts included in this category** | **Papers that include 1^st^ order constructs** | **Papers that include 2^nd^ order constructs** |
| **6. Mastering guideline-concordant care**  Guidelines` significance decreases with increased experience and knowledge. Whilst less experienced clinicians are dependent on the guidelines; senior doctors tend to be more sceptical towards it. Senior clinicians recognise their responsibility in ensuring that guidelines are followed, but they rationalise non-compliance by their autonomy and the need to adjust treatment to individual patients’ clinical situation. | - Distribution & accessibility | 8 | 5 |
|  | - Clarity & interpretation | 6, 13 | 4, 13 |
|  | - Content & agreement | 2, 5, 8, 10, 13 | 8 |
|  | - One size doesn`t fit all | 5, 13 | 8, 10, 13 |
|  | - Familiarity, awareness & acceptability | 2, 3, 5, 8, 10, 13 | 4, 5, 6, 8, 10, 12, 13 |
|  | - Need for evidence-based guidelines | 8 | - |
|  | - Guidelines are only an instrument | - | 3, 8 |
|  | - Scepticism around guidelines | 5, 13 | 3, 8, 10 |
|  | - Stringency of applying guidelines | 8, 13 | 2, 8, 10 |
|  | - Guidelines give a sense of security | 5, 8 | 2, 4, 8, 13 |
| **7. Patient-doctor relationship**  The dynamics of patient-doctor relationship are driven by family expectations (actual and perceived) , fear of lawsuit and inadequate communication. Fear of patient complaints result in low threshold for antibiotic prescribing in hospitals. This emerging culture of consumerism plays a significant role in deviating from best practice and hinders use of clinical guidelines. | - Patient demands | 3, 5, 6, 9, 15 | 3, 5, 9, 12, 15 |
|  | - Perceived patient preferences | 5, 9, 11, 13 | 3, 5, 9, 11 |
|  | - Keeping patients amused | 3, 5, 6 | 6, 9, 12 |
|  | - Patient health education | 5, 6 | 5, 6, 15 |
|  | - Patient learned behaviours | 5 | - |
|  | - Shared decision-making | 6, 9 | 5, 6 |
|  | - Consumerism and `complaints culture` | 5, 6, 9 | 9, 15 |
|  | - Fear of lawsuit | 6, 9, 15 | 6, 9 ,15 |
|  | - Keeping family happy | 3 | 2, 3, 11, 12 |
| **8. Learning the medical rite of passage**  A passage occurs through years of `learning to doctor`, acquiring knowledge and experience to become independent decision-maker able to tolerate greater risks. Being able to progress through medical career whilst maintaining a sense of competence is achieved by accumulation of clinical experience. When faced with clinical uncertainty, junior clinicians experience anxiety and tend to focus on the short-term outcomes. The reassurance of prescribing antibiotics, `just in case` there is an infection is preferred. | - Knowledge and experience | 2, 3, 4, 6, 8, 9, 11, 12, 13 | 2, 3, 4, 6, 8, 9, 11, 12, 13 |
|  | - Lack of confidence | 3, 4, 6, 9, 11, 13 | 3, 9, 11 |
|  | - Erring on the side of caution | 3, 6, 9, 11, 12, 15 | 6, 8, 11, 12, 13, 15 |
|  | - `Get them through the night` | 3, 9, 13 | - |
|  | - De-escalation anxiety | 9, 13 | 6, 9, 12, 13 |
|  | - Being able to defend decisions | 3, 4, 5, 6, 9, 11, 13 | 6, 9, 11, 12, 13 |
|  | - Fear of being criticised | 3, 4, 6, 11 | 3, 6, 10, 11, 15 |
|  | - Learning to be independent | 13 | 3, 10 |
|  | - Fear of missing something | 2, 3, 6, 11, 12, 15 | 3, 6, 9, 11, 12, 13, 15 |
|  | - Sense of competence | 11, 13, 15 | 6, 10, 11, 12, 13, 15 |
| **9. Suboptimal prescribing is a logical choice**  Antibiotics are considered a `peripheral thing` and of limited concern at the bedside, and the threat of AMR is a theoretical problem, which is morally and professionally important, but not necessarily practical in the hospital. Sub-optimal antibiotic prescribing is therefore a logical choice within the hospital. The threat of resistance is an abstract problem, as compared to the practical issue of the patient care. Over-treatment of antibiotics is therefore more favourable than the potential for adverse immediate patient outcomes and losing professional reputation. | - Narrow-spectrum antibiotics not considered effective | 2, 15 | 2, 8, 12, 13, 15 |
|  | - Antibiotics are a peripheral thing | 3, 9 | 6, 9 |
|  | - Overtreatment due to trivialisation | 3, 9, 12, 13 | 2, 5, 12 |
|  | - Mythical properties of IV antibiotics | 9 | 9, 12 |
|  | - Anxiety leads to generosity of treatment | 2, 3, 6, 9, 11, 12, 13, 15 | 2, 3, 6, 8, 9, 11, 12, 15 |
|  | - Clinical uncertainty | 2, 3, 6, 11, 12, 13 | 2, 3, 4, 5, 6, 8, 11, 12 |
|  | - Low threshold for initiation of antibiotics | 2, 3, 5, 6, 9, 11, 12, 13, 15 | 5, 6, 11, 12, 15 |
|  | - Guidelines at odds with the bedside evidence | 3, 10, 11, 12, 13 | 2, 9, 10, 11, 13, 15 |
|  | - AMR awareness not practical at the bedside | 3, 6, 15 | 2, 3, 15 |
|  | - Benefits of antibiotics outweigh the risks | 6, 12, 13, 15 | 3, 5, 6, 9, 11, 12, 15 |
|  | - Therapeutic powerlessness | 6, 12 | 12, 15 |
|  | - `Wait and see approach` | 2, 5 | 2, 5, 8, 13, 15 |
| **10. Benevolence and the emotional prerogative**  Antibiotic prescribing is an `act of kindness` driven by the hope of improving patient condition or at least providing a ` beacon of hope`. Prescribing antibiotics treatment is seen as confirmation of the doctor’s role, a trigger that at least something was done. Not to do anything is much more difficult. Immediate patient improvement means `good doctoring` even if it is at the cost of increasing AMR. | - Professional identity | 3, 11 | 3, 11, 12 |
|  | - Doing the `right thing` | 5, 6, 11, 12, 13 | 2, 3, 4, 11, 12, 13, |
|  | - Need to `do something` | 3, 5, 12, 13 | 3, 5, 12, 15 |
|  | - Being seen as a good doctor | 3, 11 | 3, 11 |
|  | - Not going home empty handed | 3, 5 | 3 |
|  | - Prescribing antibiotics for placebo effect | 3, 5 | 5 |
|  | - Beacon of hope | - | 12 |
|  | - Providing optimal care | 5, 6, 12, 15 | 2, 3, 10, 13, 15 |
| **11. Clinical inertia and transmission of habit**  Less experienced clinicians are vulnerable the norms of `generous` prescribing environment and experience pressure to conform to the current norms of practice. They feel stuck in the system dominated by prescribing trends. Certain clinicians prefer specific combinations or options of antibiotics. The idea of contesting or challenging existing practices of seniors is not attractive and causes additional stress. Being in conflict with opinion leaders is more difficult than giving in. | - Path of least resistance | 6, 9, 10, 12, 13 | 3, 6, 10, 12, 15 |
|  | - Not ready to change | 3 | 2, 10 |
|  | - Stuck in the healthcare system | 6, 9 | - |
|  | - Prescribing antibiotics is just a tick box exercise | 5, 9 | - |
|  | - Inability to make a diagnosis drives prescribing | 11 | 5, 11, 12 |
|  | - Knee-jerk reactions | 3, 6, 9, 12, 13 | 3, 5, 12 |
|  | - Mindlines | 3, 6, 9 | 3, 5 |
|  | - Prescribing trends | 3, 4, 6, 9, 11, 10, 12 | 3, 5, 6, 11 |
|  | - Ripple effects of hierarchical structures | 3, 6, 9, 10, 13 | 3, 5, 6, 9, 10, 12 |
|  | - Opposing the system causes stress | 6, 9, 12 | 3, 12, 15 |
| **12. Internalisation of peer driven practice**  Social norms of medical culture subconsciously influence prescribing behaviour of junior doctors. Antibiotic prescribing decisions are not entirely based on reason but are driven by the recognition of how ‘others do it’. The practice of colleagues is passively internalised and then subconsciously reproduced. | - Continuing the status quo | 3, 6, 9, 10 | 3, 6, 9, 10 |
|  | - Blind spots & subconscious influences | 3, 6 | 3, 6, 13 |
|  | - Hidden reasoning | 3, 6, 10, 13 | 3, 6, 10, 12, 13 |
|  | - Autonomy | 8, 9, 10 | 9, |
| **13. Learning rules of the game**  Within the medical culture, antibiotic prescribing practice is governed by a set of rules, where behaviour, attitudes and opinions of senior clinicians strongly influence practice of junior doctors. Learning the `rules of the game` of the hospital and securing professional credibility within the hierarchical team structures translates to becoming ‘competent’. | - Hierarchy | 3, 6, 9, 10, 11, 13 | 3, 4, 5, 6, 9, 10, 11, 13 |
|  | - Role models | 3, 11 | 3, 5, 6, 9, 10, 11, 13 |
|  | - Social game | 4, 6, 9, 11 | 3, 11 |
|  | - Apprenticeship model of learning | 3, 11 | 3, 10, 11 |
|  | - Avoiding confrontation | 6, 9, 10 | 3, |
|  | - Fraternal obligation | 6, 11 | 3, 6, 11 |
|  | - Maintaining professional credibility | 6, 11 | 3, 6, 11, 15 |
| **14. Managing interpersonal relationships**  Managing inter-professional relationships within the hospital culture is not straightforward. Competing dynamics mean that junior doctors often experience conflict and feel like a `piggy in the middle`. They must learn to decide whose opinion to follow in their daily practice. Negotiating prescribing decisions can be difficult at times, especially if there is an influence from various authoritative figures, including own clinical team versus other specialties. | - Us vs they | 10, 13 | 10 |
|  | - Conflicting opinions | 4, 5, 8, 9, 10, 13 | 4, 10 |
|  | - Piggy in the middle | 4, 5 | 4 |
|  | - Dynamics between different specialties | 5, 10, 13 | 10 |
|  | - Support and advice from colleagues | 8, 9, 10, 11, 13 | 2, 3, 4, 6, 8, 10, 11, 13 |
|  | - Competing interests | 5, 10 | 10 |
|  | - Poor communication due to trivialisation | 5 | 12 |
|  | - Advice from ID specialists | 2, 5, 8, 9, 10 | 2, 8, 10 |
|  | - Influence of microbiologists | 4, 8 | 8, 10, 13 |
|  | - Role of clinical pharmacists | 9, 13 | 6, 13 |
|  | - Nurses` influence | - | 12 |
| **15. Perceived facilitators to antimicrobial stewardship**  Within the busy hospital environment, support and regular feedback from more experienced clinicians and the opportunity to work independently are valued by junior doctors. A collaborative culture fostering multidisciplinary approach and normalisation of the role of other specialists within decision-making process are crucial to aid improvements. The quality of inter-professional relationships between clinicians is key to achieving change. Equal distribution of the responsibility of prescribing decision beyond that of the senior consultant in charge of the patient needs to be addressed. | - Patient and provider education | 4, 5 | 2, 5, 6, 8, 9, 10, 15 |
|  | - Supervision & performance feedback mechanisms | 4, 11 | 4, 5, 6, 8, 11 |
|  | - Formal Teaching | 4, 5, 8, 11 | - |
|  | - Induction | - | 4 |
|  | - Good handovers | - | 4, 8 |
|  | - Clinical decision-making support | 5 | 5 |
|  | - Improved diagnostic testing | 5 | 5, 6 |
|  | - Written materials & signposting | - | 4, 8 |
|  | - Raised awareness of AMR | - | 2, 15 |
|  | - Healthcare system that supports restrictive prescribing | 5, 9 | 2, 3, 4, 5, 9, 13 |
|  | - Multidisciplinary approach and support from senior colleagues | 3, 4, 6, 8 | 4, 5, 6, 8, 9, 10, 13 |
|  | - Tailored guidelines | - | 5 |
|  | - Approval system | - | 10 |
|  | - Distribution of responsibility | - | 13 |
| **16. Organisational constraints that prevent optimal treatment**  The opportunities to learn and refine the craft of prescribing within the complex ward-based environment are scarce due to fast-paced hospital environment and time pressures. Limited senior support and feedback on prescribing contributes to communication problems and poses a challenge to continuity of patient care.  Variability in practice and lack of continuity or a follow-up of prescribing decisions due to doctors` rotations and patients being moved between wards, error-prone handovers, unclear instructions and lack of follow-up all contribute a lack of ownership of prescribing decisions. | - Time pressures and fast-paced environment | 3, 4, 5, 6, 9, 12, 13 | 2, 4, 5, 12 |
|  | - Lack of follow-up | 2, 5, 6, 8, 9, 15 | 4, 5, 6, 8, 9, 13, 15 |
|  | - Continuity of care | 2, 5, 6, 9, 13 | 4, 5, 6, 9, 12, 13 |
|  | - Poor communication | 6 | 4, 13 |
|  | - Out of hours working | 13 | 4, 8, 11, 12 |
|  | - Variations in practice | 6, 11 | 4, 6, 9, 11, 12, 13 |
|  | - Feeling pressurised | 3, 4 | 4 |
|  | - Poor handovers | 4, 11 | 4, 9, 11 |
|  | - External institutional influences | 4, 15 | 2, 12, 15 |
|  | - Navigating a patchwork system of resource allocation | 4, 5, 8 | 2, 5, 8 |
|  | - Lack of induction and formal teaching | 2, 4, 8 | 4 |
|  | - Lack of support from seniors | 11 | 4, 9, 11, 13 |
|  | - Unclear responsibilities | - | 4, 13 |
| **17. Factors influencing antibiotic decision-making**  The fear of patient deteriorating linked with the expectation placed on juniors to prescribe antibiotics as soon as possible can often lead to overtreatment. When patient diagnostics are inconclusive, or in circumstances of clinical uncertainty when infection is difficult to distinguish from other disorders, it feels safer to prescribe antimicrobials than not. | - Severity of disease determines prescribing | 6, 8, 13 | 8, 13 |
|  | - De-escalation based on micro results/patient`s improvement | 9 | - |
|  | - Consideration of AMR | 2, 3, 5, 6, 11, 15 | 2, 3, 11, 15 |
|  | - Dilemma of prescribing vs non-prescribing | 6, 11, 15 | 11, 15 |
|  | - Clinical picture is a good indicator of patient status | 13 | 6, 8, 12, 13 |
|  | - Antibiotic decisions not prioritised | - | 9, 15 |

**References:**

[1] Cortoos P-J, De Witte K, Peetermans WE, Simoens S, Laekeman G. Opposing expectations and suboptimal use of a local antibiotic hospital guideline: a qualitative study. J Antimicrob Chemother. 2008;62:189–95. https://doi.org/10.1093/jac/dkn143.

[2] Bjorkman I, Berg J, Roing M, Erntell M, Lundborg CS, I. B, et al. Perceptions among Swedish hospital physicians on prescribing of antibiotics and antibiotic resistance. Qual and Safety in Health Care. 2010;19(6):e8. https://doi.org/10.1136/qshc.2008.029199.

[3] Broom A, Broom J, Kirby E. Cultures of resistance? A Bourdieusian analysis of doctors’ antibiotic prescribing. Soc Sci Med. 2014;110:81–8. https://doi.org/10.1016/j.socscimed.2014.03.030.

[4] Mattick K, Kelly N, Rees C. A window into the lives of junior doctors: narrative interviews exploring antimicrobial prescribing experiences. J Antimicrob Chemother. 2014;69:2274–83. https://doi.org/10.1093/jac/dku093.

[5] May L, Gudger G, Armstrong P, Brooks G, Hinds P, Bhat R, et al. Multisite exploration of clinical decision making for antibiotic use by emergency medicine providers using quantitative and qualitative methods. Infect Control Hosp Epidemiol. 2014;35:1114–25. https://doi.org/10.1086/677637.

[6] Livorsi D, Comer A, Matthias MS, Perencevich EN, Bair MJ. Factors Influencing Antibiotic-Prescribing Decisions Among Inpatient Physicians: A Qualitative Investigation. Infect Control Hosp Epidemiol. 2015;36:1065–72. https://doi.org/10.1017/ice.2015.136.

[7] Livorsi D, Comer AR, Matthias MS, Perencevich EN, Bair MJ. Barriers to guideline-concordant antibiotic use among inpatient physicians: A case vignette qualitative study. J Hosp Med. 2016;11:174–80. https://doi.org/10.1002/jhm.2495.

[8] Skodvin B, Aase K, Charani E, Holmes A, Smith I, et al. An antimicrobial stewardship program initiative: a qualitative study on prescribing practices among hospital doctors. Antimicrob Resist Infect Control. 2015;4:24. https://doi.org/10.1186/s13756-015-0065-4.

[9] Broom J, Broom A, Adams K, Plage S. What prevents the intravenous to oral antibiotic switch? A qualitative study of hospital doctors’ accounts of what influences their clinical practice. J Antimicrob Chemother. 2016;71:2295–9. https://doi.org/10.1093/jac/dkw129.

[10] Broom J, Broom A, Plage S, Adams K, Post JJ. Barriers to uptake of antimicrobial advice in a UK hospital: a qualitative study. J Hosp Infect. 2016;93:418–22. https://doi.org/10.1016/j.jhin.2016.03.011.

[11] Broom A, Broom J, Kirby E, Adams J. The social dynamics of antibiotic use in an Australian hospital. J Sociol. 2016;52:824–39. https://doi.org/10.1177/1440783315594486.

[12] Eyer MM, Lang M, Aujesky D, Marschall J. Overtreatment of asymptomatic bacteriuria: a qualitative study. J Hosp Infect. 2016;93:297–303. https://doi.org/10.1016/j.jhin.2016.04.007.

[13] Rawson TM, Charani E, Moore LSP, Hernandez B, Castro-Sanchez E, Herrero P, et al. Mapping the decision pathways of acute infection management in secondary care among UK medical physicians: a qualitative study. BMC Med. 2016;14:208. https://doi.org/10.1186/s12916-016-0751-y.

[14] Sedrak A, Anpalahan M, Luetsch K. Enablers and barriers to the use of antibiotic guidelines in the assessment and treatment of community-acquired pneumonia—A qualitative study of clinicians’ perspectives. Int J Clin Pract. 2017;71:e12959. <https://doi.org/10.1111/ijcp.12959>.

[15] Broom J, Broom A, Kirby E, Gibson AF, Post JJ. Individual care versus broader public health: A qualitative study of hospital doctors’ antibiotic decisions. Infect Dis Heal. 2017;22:97–104. https://doi.org/10.1016/j.idh.2017.05.003.
