# Supplementary material for: Understanding the complexities of antibiotic prescribing behaviour in acute hospitals: a systematic review and meta-ethnography
Source: Arch Public Health. 2021 Jul 23;79:134. doi: 10.1186/s13690-021-00624-1 (PMC8299683; doi:10.1186/s13690-021-00624-1)
Supplement: Supplementary file 3 — Additional file 3. Key emerging themes with exemplar quotes. [file 13690_2021_624_MOESM3_ESM.doc]

**Additional file 3: Key emerging themes with exemplar quotes**

| **Theme** |  | **Exemplar quotes** |
| --- | --- | --- |
| 1. Loss of ownership of prescribing decisions | Q1 | “It [antibiotic choice] depends on the time of day that you’re admitting the patient, and on a lot of other factors, whether you can ask your advanced trainee or your boss…sometimes you’re busy. you’ve kind of heard half of the story, and you haven’t fully done everything yourself, and it sounds like a good going infection, so you prescribe.”  *- Non-consultant, Oncology, Female, Australia* [1] |
|  | Q2 | “So I feel quite, I wouldn’t say disempowered, but I feel like the seniors make most of the decisions. So I’m quite reluctant to make any decisions about [de-escalating] antibiotics.” *- On-rotation, Gastroenterology, UK* [2] |
|  | Q3 | “One thing about emergency is that we treat people at their initial presentation, and unless we make the effort to follow-up someone through the ward, we actually don’t know what happens to them.”  - *Emergency Department, Australia* [3] |
|  | Q4 | “If a patient moves from one ward to the other and decision has been taken to give antibiotics, then there should also have been decided what the duration of therapy should be! Sometimes, it`s very difficult to retrieve what the indication was!” *- Consultant, Internal Medicine, Belgium* [4] |
| 2. Tension between individual care and broader public health concerns | Q5 | “It [the problem of antibiotic resistance] is always there at the back of your mind, but then sometimes when you are faced with a particular situation, you're stuck between trying to think on the global way of trying to reduce broad-spectrum antibiotic use and all that versus trying to make sure you don't miss a bug by going too narrow.”  - *Resident, USA* [5] |
|  | Q6 | “Like most things in medicine, especially when it comes down to thinking about, as you said, a global context or how much things cost or these big picture things, but we really are taught that we should be looking at the patient in front of us and doing what is best for that patient.” - *Clinician*, *Australia* [6] |
|  | Q7 | *“It’s very unusual that anyone would actually explain to you what they’re thinking. I think I’ve had one explanation which was like a ray of sunshine” - On-rotation, Renal UK* [2] |
| 3. Evidence-based practice versus bedside medicine | Q8 | “We are trained to do something and fix something, so to not do anything is probably the hardest guideline to follow” *- Resident, USA* [7] |
|  | Q9 | “I know this is not what microbiologists would say, but in my mind you like to feel like you are doing something...and that’s why you give them some intravenous antibiotics…and I think it makes everyone feel better whether it’s the patient and more significantly the doctor” - *Consultant, General Internal Medicine, UK* [2] |
|  | Q10 | “Just the thought of not covering some resistant organism or more pathogenic organism, even though I do not have any definitive objective evidence, always makes me quite anxious.” - *Consultant, USA* [5] |
|  | Q11 | “I think in my mind it is you know, ‘Will my patient do as well? Will they respond?’ .. the major qualm I have about someone else looking at me is not ‘Oh, you didn’t prescribe a broad enough antibiotic.” - *Geriatrics Consultant, Australia* [8] |
|  | Q12 | “With the complaints culture, and the amount of litigation going on, I think a lot of doctors are afraid to stand up and say ‘no, you really don’t need antibiotics’, or ‘there’s really no indication’, or ‘you’re alright for now, let’s just wait and see what the blood test shows’, or things like that.” *- Medical, Non-Consultant, UK* [9] |
| 4. Diverse priorities between different clinical teams | Q13 | “I think it’s because the risk of a person getting an infection comes down onto them [surgeons] personally, not antimicrobial stewardship, not pharmacy, not ID, but them personally. So, if their patient gets an infection in a weeks’ time, they’re the one getting into trouble, and they’re the one the patient’s going to blame.” *- Clinician, Australia* [6] |
|  | Q14 | “…sometimes those guidelines do not cover all the bases, and you still need to do what you think is best for the patient...” *- Attending, USA* [7] |
|  | Q15 | *“And it’s certainly in one trust I worked [as junior doctor] where the bronchiectasis specialist and the microbiologist often clashed overuse of gentamicin. I was frequently caught in that.” - Medical Consultant, Male, UK* [10] |
|  | Q16 | *“I think if it isn’t clearly in the guideline or I am not sure, if it doesn’t easily fit into the guideline, I am going to say [to my juniors], okay speak to microbiology and see what they think” - Respiratory Consultant, UK* [2] |
|  | Q17 | “Concerning antibiotic treatment, we follow a simple algorithm, but when things get complicated, we collaborate with the ID-specialists, and intensive care doctors, of course” - Gastro-Surgery Consultant, Norway [11] |

**References:**

[1] Broom A, Broom J, Kirby E. Cultures of resistance? A Bourdieusian analysis of doctors’ antibiotic prescribing. Soc Sci Med. 2014;110:81–8. https://doi.org/10.1016/j.socscimed.2014.03.030.

[2] Rawson TM, Charani E, Moore LSP, Hernandez B, Castro-Sanchez E, Herrero P, et al. Mapping the decision pathways of acute infection management in secondary care among UK medical physicians: a qualitative study. BMC Med 2016;14:208. https://doi.org/10.1186/s12916-016-0751-y.

[3] Sedrak A, Anpalahan M, Luetsch K. Enablers and barriers to the use of antibiotic guidelines in the assessment and treatment of community-acquired pneumonia—A qualitative study of clinicians’ perspectives. Int J Clin Pract. 2017;71:e12959. https://doi.org/10.1111/ijcp.12959.

[4] Cortoos P-J, De Witte K, Peetermans WE, Simoens S, Laekeman G. Opposing expectations and suboptimal use of a local antibiotic hospital guideline: a qualitative study. J Antimicrob Chemother. 2008;62:189–95. https://doi.org/10.1093/jac/dkn143.

[5] Livorsi D, Comer A, Matthias MS, Perencevich EN, Bair MJ. Factors Influencing Antibiotic-Prescribing Decisions Among Inpatient Physicians: A Qualitative Investigation. Infect Control Hosp Epidemiol. 2015;36:1065–72. https://doi.org/10.1017/ice.2015.136.

[6] Broom J, Broom A, Kirby E, Gibson AF, Post JJ. Individual care versus broader public health: A qualitative study of hospital doctors’ antibiotic decisions. Infect Dis Heal. 2017;22:97–104. https://doi.org/10.1016/j.idh.2017.05.003.

[7] Livorsi D, Comer AR, Matthias MS, Perencevich EN, Bair MJ. Barriers to guideline-concordant antibiotic use among inpatient physicians: A case vignette qualitative study. J Hosp Med. 2016;11:174–80. https://doi.org/10.1002/jhm.2495.

[8] Broom A, Broom J, Kirby E, Adams J. The social dynamics of antibiotic use in an Australian hospital. J Sociol. 2016;52:824–39. https://doi.org/10.1177/1440783315594486.

[9] Broom J, Broom A, Adams K, Plage S. What prevents the intravenous to oral antibiotic switch? A qualitative study of hospital doctors’ accounts of what influences their clinical practice. J Antimicrob Chemother. 2016;71:2295–9. https://doi.org/10.1093/jac/dkw129.

[10] Broom J, Broom A, Plage S, Adams K, Post JJ. Barriers to uptake of antimicrobial advice in a UK hospital: a qualitative study. J Hosp Infect. 2016;93:418–22. https://doi.org/10.1016/j.jhin.2016.03.011.

[11] Skodvin B, Aase K, Charani E, Holmes A, Smith I, et al. An antimicrobial stewardship program initiative: a qualitative study on prescribing practices among hospital doctors. Antimicrob Resist Infect Control. 2015;4:24. https://doi.org/10.1186/s13756-015-0065-4.
